# Supplementary material for: A qualitative study of bereavement support volunteers’ views and experiences on an online Acceptance and commitment therapy-based (ACT) training programme
Source: PLoS One. 2025 Dec 8;20(12):e0337321. doi: 10.1371/journal.pone.0337321 (PMC12685200; doi:10.1371/journal.pone.0337321)
Supplement: S1 File — (PDF) [file pone.0337321.s001.pdf]

THE UNIVERSITY of EDINBURGH

Cruse Scotland
Bereavement Support

FUNDED BY
Marie Curie
Care and support through terminal illness

mygrief my way

Support Volunteer Training: Session One

Dr. David Gillanders
University of Edinburgh

Starting from where you are at…

- Different skills and experiences
- Mutual respect and compassion: safety
- Discussion based / reflective / experiential
- Not just a presentation / facts etc.

Think of a person you have worked with…

- What aspects of grieving have they struggled with?
- What have they done to deal with those difficult aspects?
- What have been the consequences?

What is the strategy?

- “It’s too painful, I need to get past it.”
- “How can I ‘move on’?”
- Sense of stuckness

The University of Edinburgh is a charitable body, registered in Scotland, with registration number SC005336

THE UNIVERSITY of EDINBURGH

The land of loss and the land of rebuilding

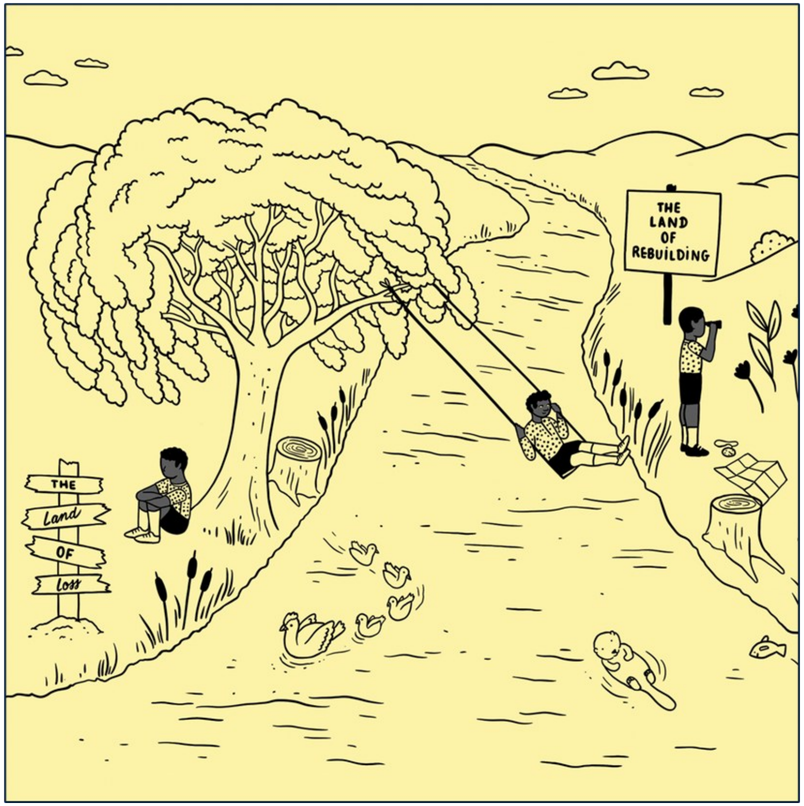

Image credit: Reprinted from the book "You will be OK" by Julie Stokes, Hachette Publishing: www.hachette.co.uk/titles/julie-stokes/you-will-be-okay/9781526363886/ under a CC BY license, with permission from Lauren Boglio: www.boglio.com, 2021.

Maybe control is part of the problem

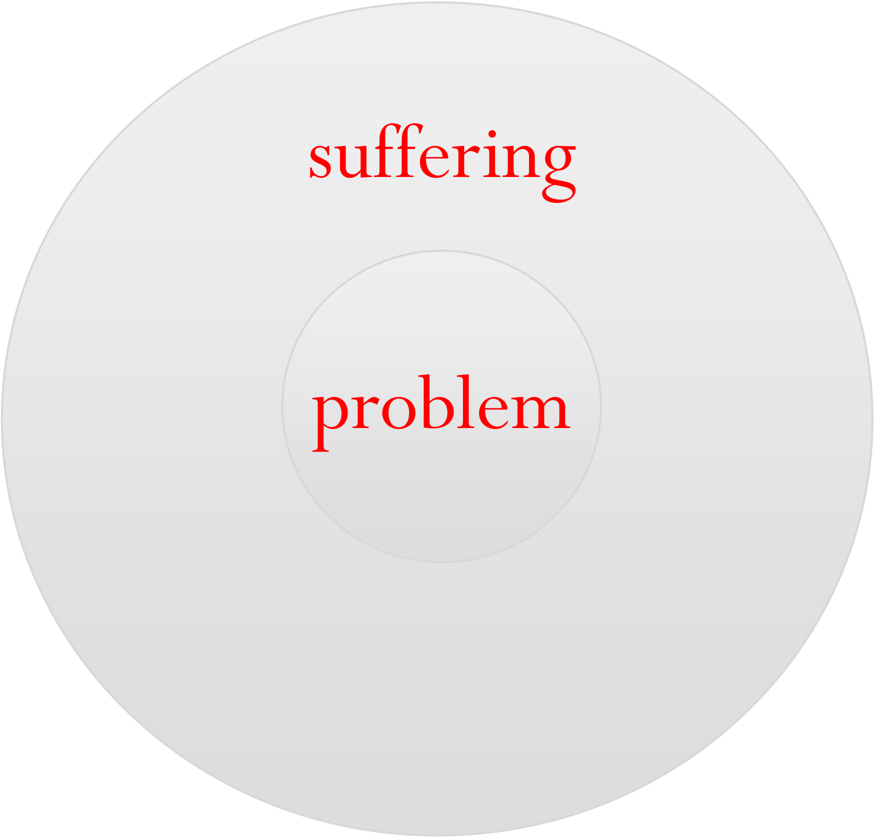

You are not broken…

- The strategy is the problem
- We need to let go of what doesn't work and do something different

The University of Edinburgh is a charitable body, registered in Scotland, with registration number SC005336

THE UNIVERSITY of EDINBURGH

Growing a life

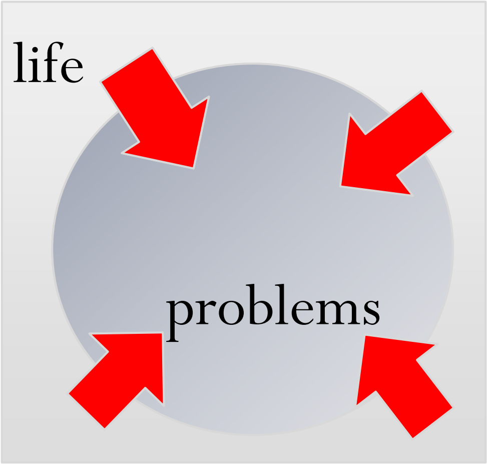

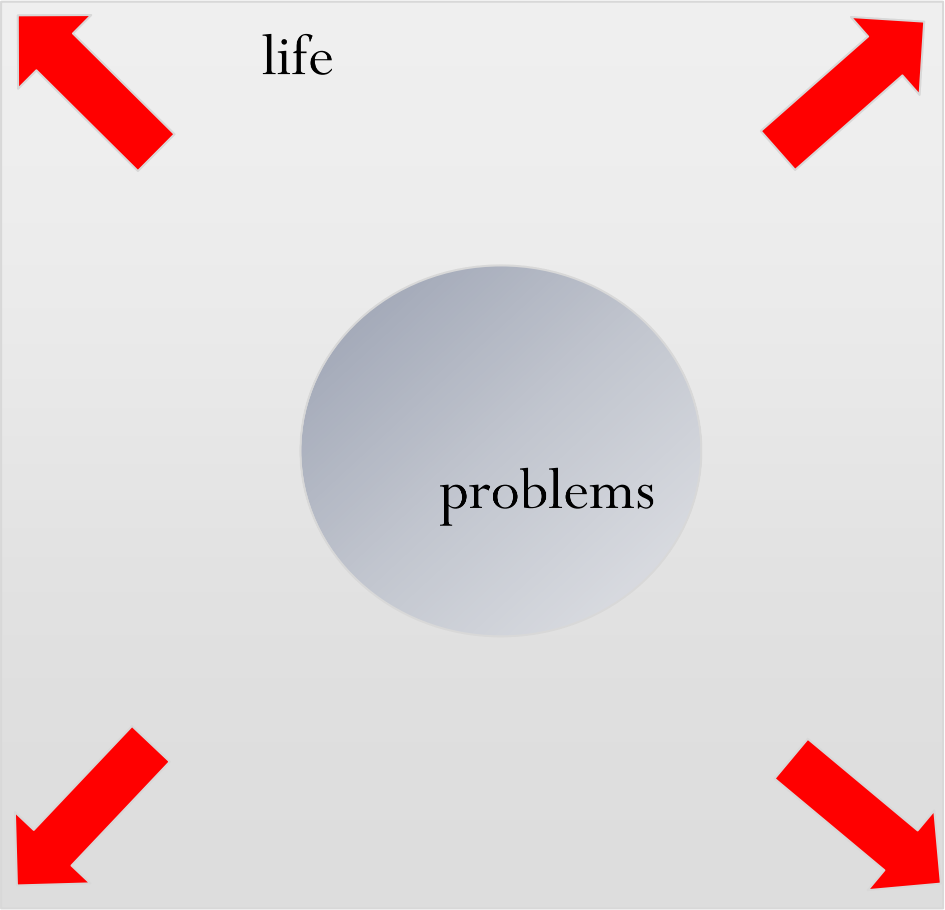

So what is Acceptance and Commitment Therapy?

- A form of cognitive behavioural therapy
- Incorporates techniques from mindfulness and compassion
- And techniques from behaviour therapy
- To help people live with difficult thoughts, feelings, sensations.

- Not about getting rid of feelings
- Not about thinking positive thoughts

- Learning skills to allow grieving to happen

The Simplified ACT Model: The Tri Flex

The ACT model: Psychological Inflexibility

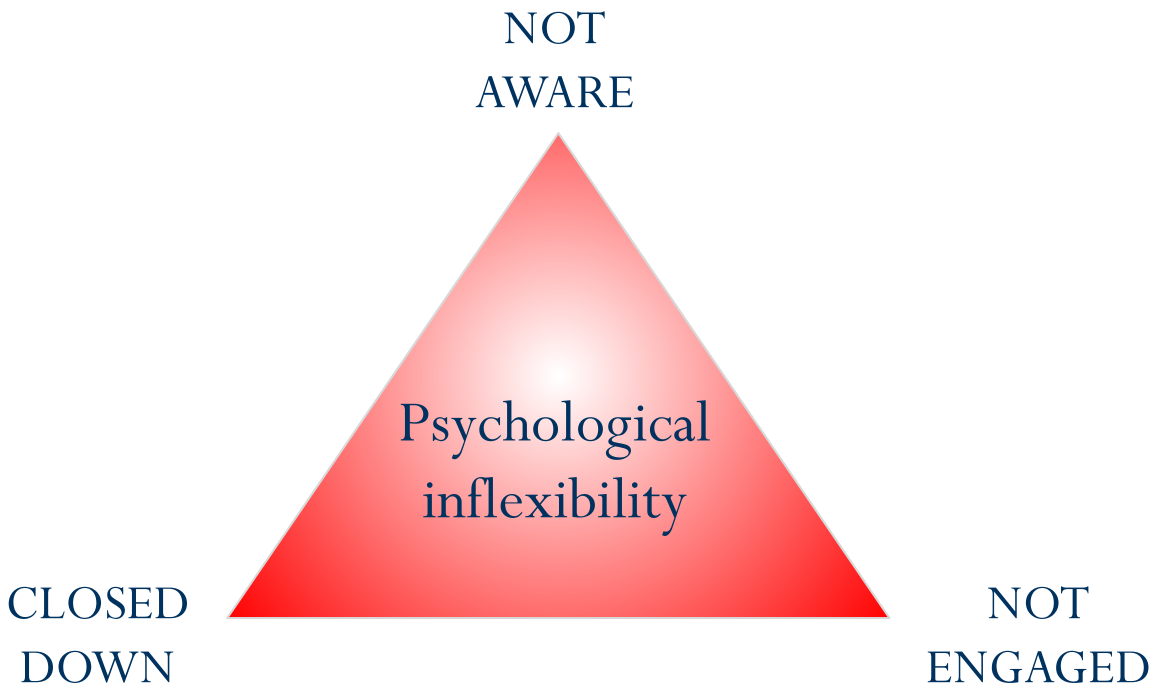

The ACT model: Psychological Flexibility

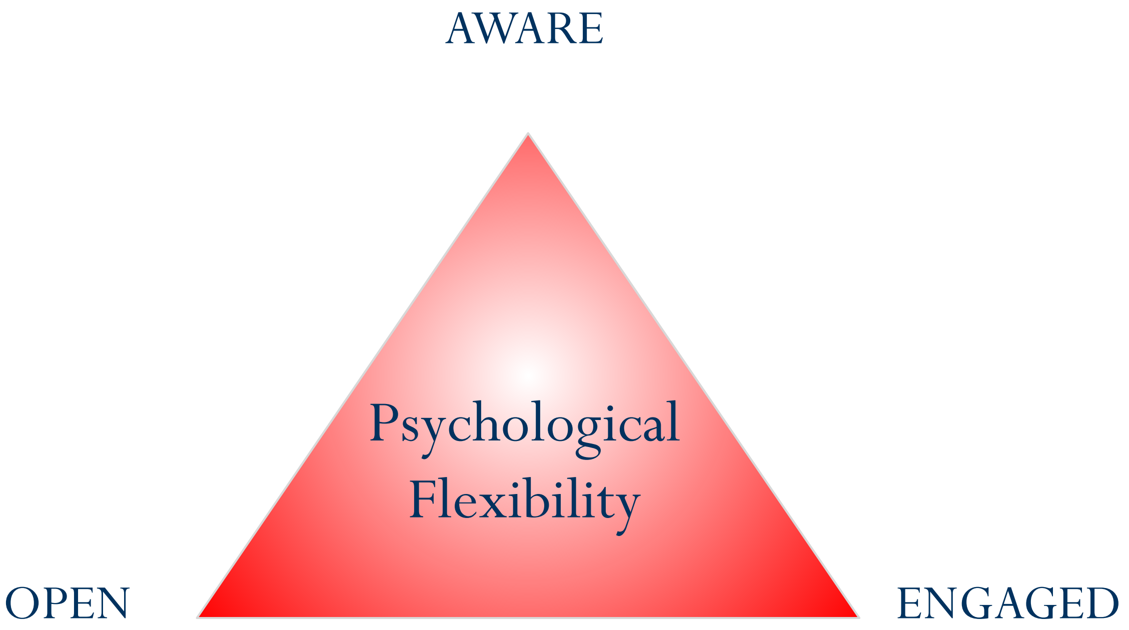

The ACT model: Psychological Flexibility

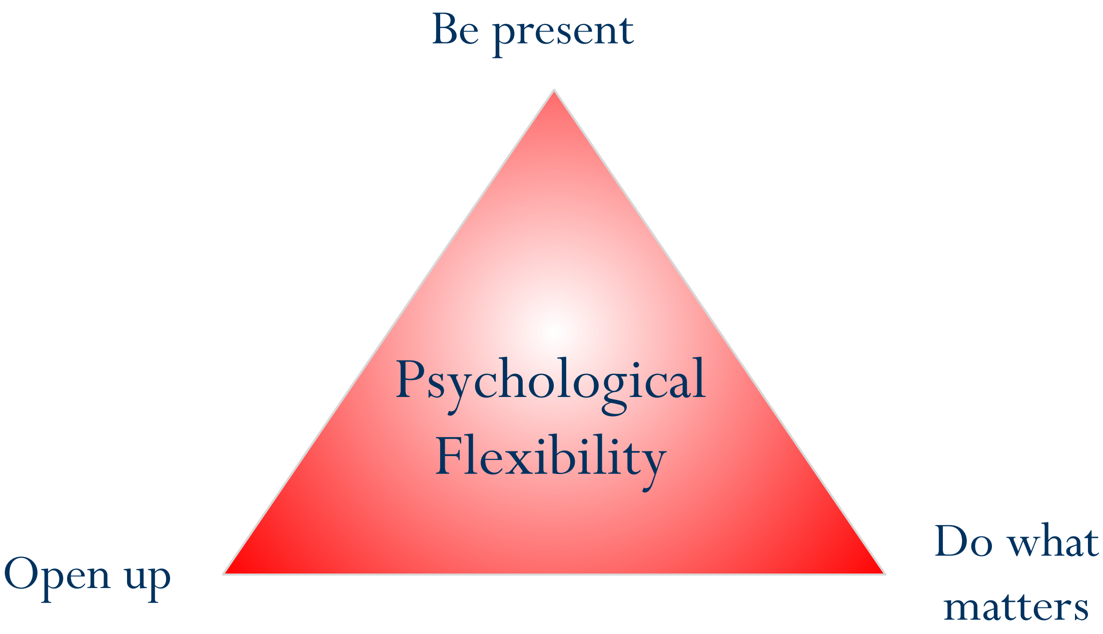

Adapting ACT for grief

Our process…

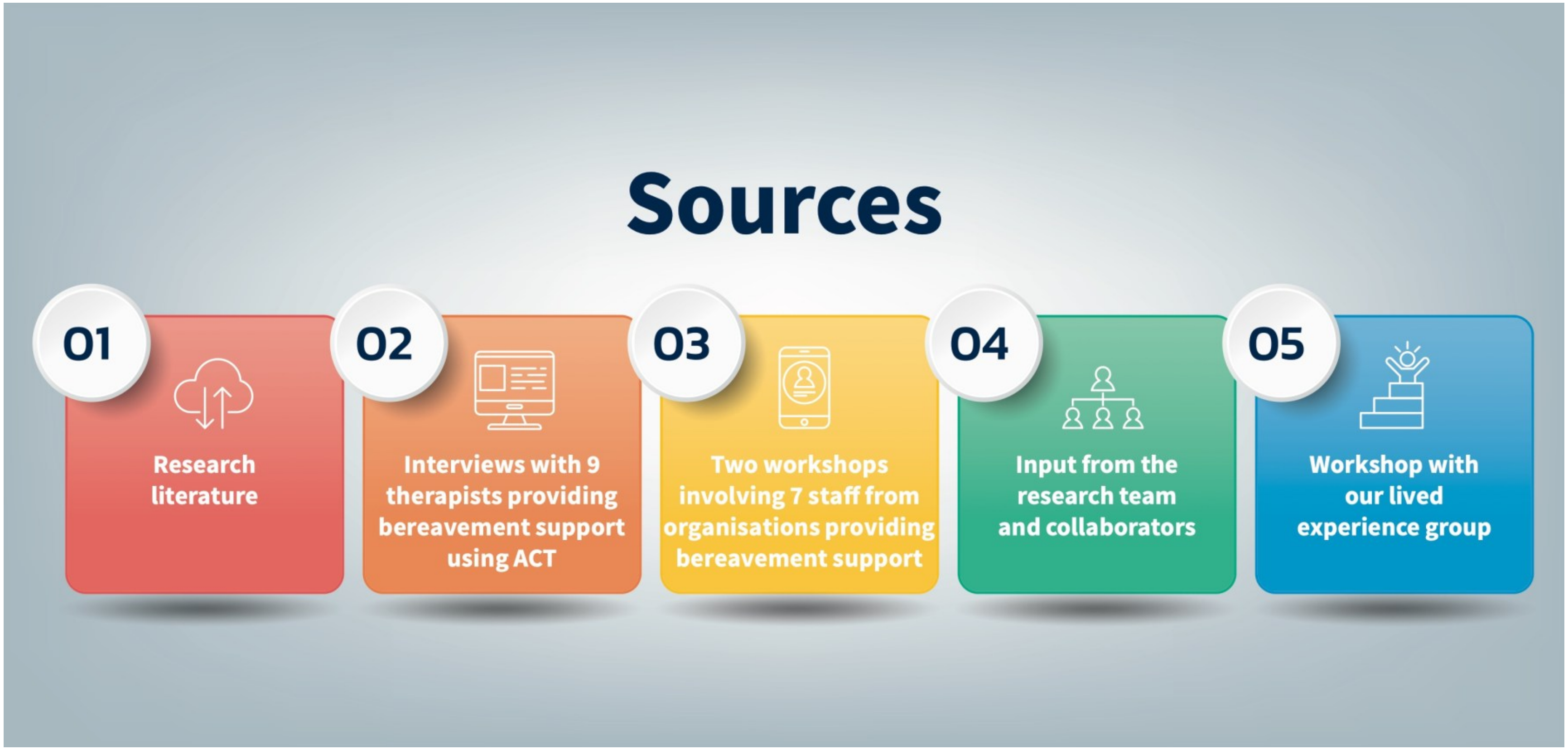

The University of Edinburgh is a charitable body, registered in Scotland, with registration number SC005336

THE UNIVERSITY of EDINBURGH

The programme theory

mygrief my way

My Grief My Way Logic model

RISK FACTORS FOR PROBLEMATIC GRIEF

SITUATIONAL
- Financial hardship
- Social isolation
- Kinship care or the care system
- Minoritised communities

CIRCUMSTANCES AROUND LOSS
- Unexpected death
- Violent death
- Death of a child
- Loss of support system
- Young age of bereaved person
- Carer
- Bereavement during COVID

INDIVIDUAL
- Past history of depression, separation anxiety or post traumatic stress disorder
- Traumatic childhood experiences
- Complex relationship with the deceased person
- Multiple losses

INDICATORS OF PROBLEMATIC GRIEF

OVERWHELMING NEGATIVE EMOTION
- Guilt, anger, fear, regret, anxiety, depression

OVERWHELMING PRACTICAL AND FINANCIAL WORRIES

LOSS OF MEANING AND PURPOSE
- Unable to move forward
- Loss of identity / identity disruption
- Spiritual crisis

BURDEN OF EXPECTATIONS
- Self / others / society
- Unsure 'how to grieve'

SELF-CRITICISM

ISOLATION AND LONELINESS
- Loss of physical contact
- Connection and intimacy
- Separate / cut-off from others

TRAUMATIC MEMORIES
- Relating to how the deceased died
- Complex relationship with deceased (unresolved)

AVOIDANCE
- Alcohol / Drugs
- Unable to go to certain places
- Emotional numbness

PHYSICAL IMPACT OF GRIEF
- Pain
- Fatigue / Exhaustion
- Sleep difficulties

INTERVENTION COMPONENTS

STORIES ILLUSTRATING COMMON STUCK POINTS WITH GRIEF
- Videos
- Audio / Podcasts
- Images
- Text

METAPHORS TO SUPPORT PERSPECTIVE TAKING

EXERCISES TO SUPPORT MINDFULNESS, AWARENESS, SELF-COMPASSION, CONNECTION WITH VALUES

ART / POETRY TO SUPPORT PRESENT MOMENT AWARENESS AND PERSPECTIVE TAKING

LINKS WITH OTHER APPROACHES
- Psychoeducation
- Compassion focused therapy
- Trauma focused
- Dual process model
- Philosophical perspectives

ACT TRAINED VOLUNTEER SUPPORT IF NEEDED

INTERVENTION FEATURES

- Easy to navigate
- Easy to access
- Feedback
- Targeted – 2 levels depending on need
- Flexible starting point
- Strategies to sustain
- Safe
- Links to further resources
- Access to trained bereavement support volunteers

BEHAVIOURAL DETERMINANTS

- Internet access
- Comfort with online intervention
- Volunteer availability if needed
- Motivation / Commitment
- Practice
- Feedback

BEHAVIOUR CHANGE MECHANISMS

PSYCHOLOGICAL FLEXIBILITY

DEVELOPING OPENNESS
- Defusion
- Acceptance

DEVELOPING AWARENESS
- Contact with present moment
- Perspective-taking

ENGAGEMENT AND COMMITTED ACTION
- Reconnecting with values and what matters
- Taking steps to do what matters

OUTCOMES

SHORT TERM

INDIVIDUAL
- Reduction in overwhelming emotion
- Increase in resilient coping response
- Increase in wellbeing
- Greater psychological flexibility
- Improved social support
- Feel better able to cope

ORGANISATIONAL
- Acceptable evidence based intervention
- Immediate support available to clients

LONG TERM

INDIVIDUAL
- Engaging in valued activities
- Reconnection with old hobbies / friendships
- Developing new hobbies / friendships
- Making space around grief – can remember their loved one without feeling overwhelmed

ORGANISATIONAL
- Able to support more people
- Cost-effective
- Offer support where there is a waiting list
- Reduce pressure on services
- Volunteers trained in evidence based approaches

Indicators of Problematic Grief

- Overwhelming negative emotions
- Avoidance
- Burden of expectations (including ‘shoulds’ and self-criticism)
- Loss of meaning or purpose
- Isolation and loneliness
- Unexpected, shocking or traumatic deaths
- Overwhelming financial and practical worries
- Physical impacts of grief

My Grief My Way Elements

Films: real stories

- Overwhelming negative emotions
- Avoidance
- Burden of expectations (including 'shoulds' and self-criticism)
- Loss of meaning
- Isolation and loneliness
- Unexpected, shocking or traumatic deaths

Understanding grief

- Normalising
- Healing
- Physical impacts
- Practical supports

Ideas & Practices

- ACT
- Compassion
- Continuing Bonds
- Dual Processing

Fitting MGMW with what you do…

- Start where you are at with listening, validating, helping them to open up
- Familiarise yourself with the My Grief My Way content
- Practice the exercises to get a feel for them
- Gently suggest elements that might be helpful
- Help them to build their skills in AWARE, OPEN, ENGAGED
- Help them troubleshoot some of the ideas and practices
- Bring any thoughts and reflections to supervision or email us

The rest of the training sessions

Training

- Session 1: Overview, what is ACT, why ACT & grief
- Session 2: Supporting AWARE & OPEN Skills
- Session 3: Supporting ENGAGED Skills
- Session 4: Using MGMW in your work

Supervision

- Three rounds of intervention development over 2024
- We will meet in small groups twice during each round

THE UNIVERSITY *of* EDINBURGH

Cruse Scotland
Bereavement Support

FUNDED BY
Marie Curie
Care and support through terminal illness

mygrief *my way*
