# Supplementary material for: A qualitative study of bereavement support volunteers’ views and experiences on an online Acceptance and commitment therapy-based (ACT) training programme
Source: PLoS One. 2025 Dec 8;20(12):e0337321. doi: 10.1371/journal.pone.0337321 (PMC12685200; doi:10.1371/journal.pone.0337321)
Supplement: S2 File — (PDF) [file pone.0337321.s002.pdf]

THE UNIVERSITY *of* EDINBURGH

Cruse Scotland
Bereavement Support

FUNDED BY
Marie Curie
Care and support through terminal illness

mygrief my way

Support Volunteer Training: Session Two

Dr. David Gillanders

University of Edinburgh

General aspects of skills shaping

MODEL

EVOKE

REINFORCE

The University of Edinburgh is a charitable body, registered in Scotland, with registration number SC005336

Reinforcing matters

- Many people neglect this
- It's really important!
- Even slightly more flexible behaviour
- Shape it, shape it, shape it
- To catch that, we need to enhance our own awareness skills

The ACT model: Psychological Flexibility

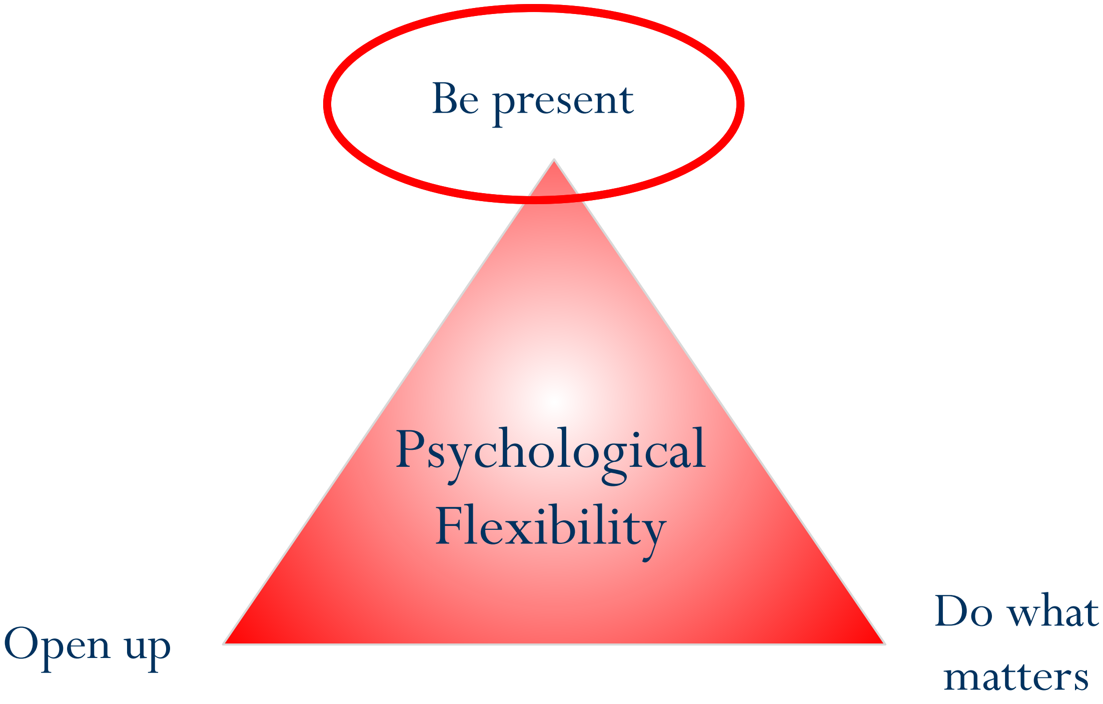

Being Present

Ways of noticing

- Formal mindfulness exercises and practice
- Informal mindfulness practices

Shaping the general skill of being present:

- What is influencing my behaviour in this moment?
- What possibilities are there for me in this moment?
- What is likely to be the most effective thing to do?

Tracking & Monitoring

- Monitoring activities:

  Diaries & Charts

- Tracking consequences

The University of Edinburgh is a charitable body, registered in Scotland, with registration number SC005336

In Session Language Cues

- In session language:
  "What do you notice as we explore this?"
  "What's pulling for your attention right now?"
  "How does this land?"

- Out of session pointing:
  "What do you think was influencing you then?"
  "What did you do when…"
  "What happened next?"
  "What do you think would happen if you were to…."

The University of Edinburgh is a charitable body, registered in Scotland, with registration number SC005336

Generalizable cues

- Could be a reminder (e.g. an alarm on a phone, a diary appointment with self)
- Can be language (e.g. 'Well noticed!")
- Could be a physical object or a visual cue

The University of Edinburgh is a charitable body, registered in Scotland, with registration number SC005336

Facilitating Openness

The ACT model: Psychological Flexibility

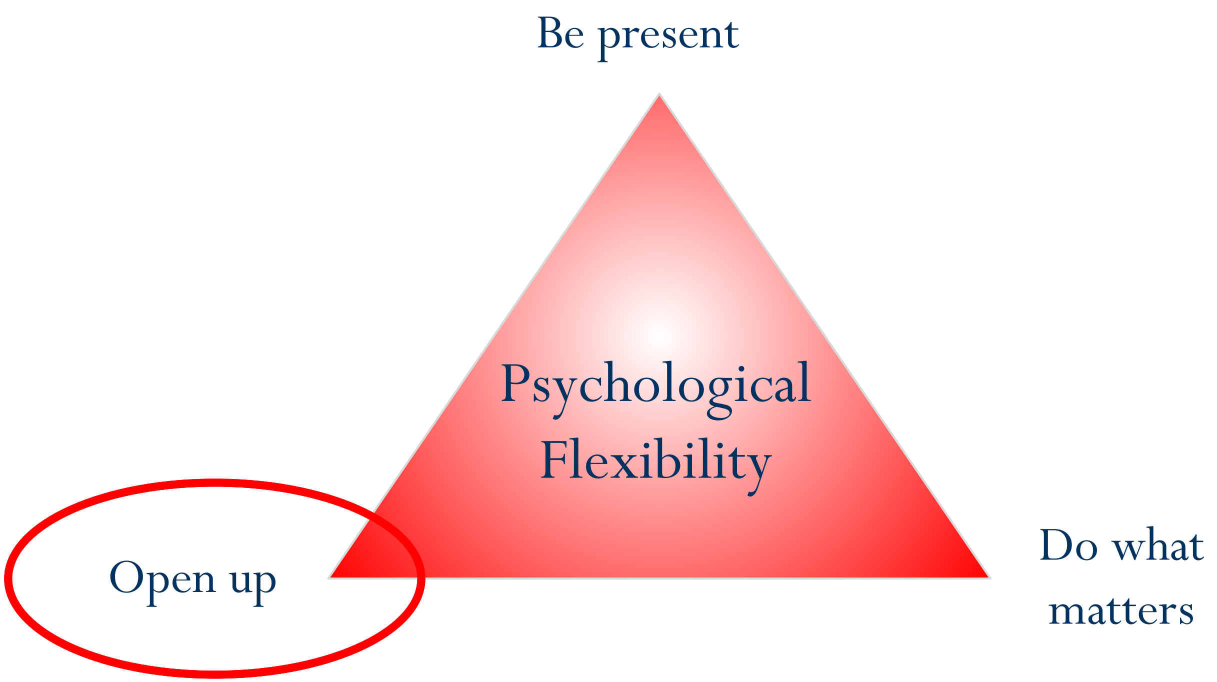

THE UNIVERSITY *of* EDINBURGH

Two Aspects of Open Skills

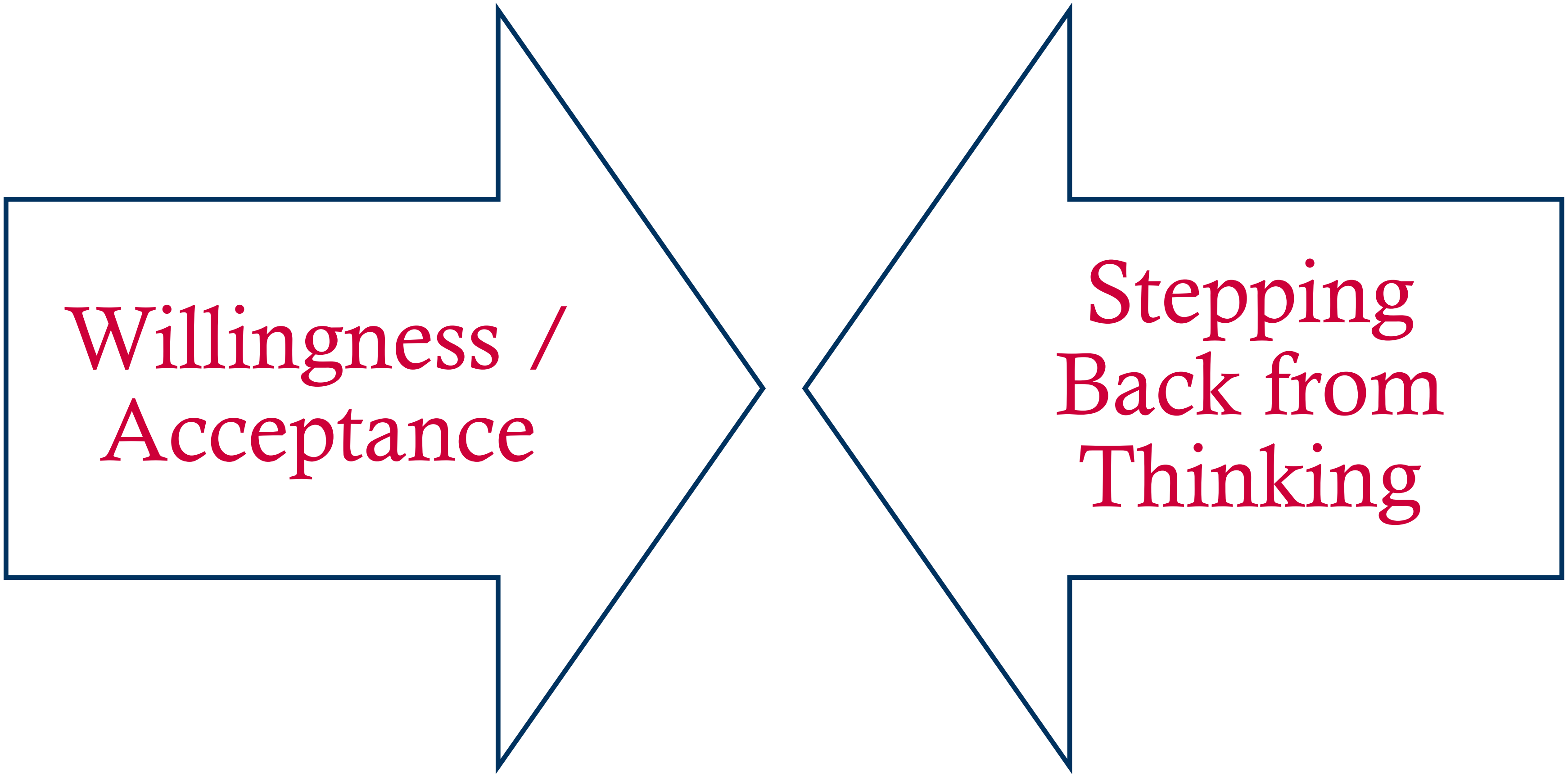

Talking about Acceptance

- ‘Acceptance’ as a double meaning
- Acceptance as a stage / state
- Acceptance as a behaviour – a choice
- Willing is not wanting or accepting or approving
- ‘Leaning in’, ‘softening’, making room’

Talking about Acceptance

- Acceptance, blame and forgiveness
- Letting yourself off the hook
- Letting others of the hook in order to free yourself
- What do you think that your loved one would want for you.

Doing Willingness

- Tracking consequences:
  - “So when you do that, what happens?”
- Physical gestures: fighting, hiding, figuring out, versus acceptance
- Stories & Metaphors:
  - flowing like water,
  - swamp and mountain

The University of Edinburgh is a charitable body, registered in Scotland, with registration number SC005336

Willingness

- Swamp and mountain metaphor

Word Games / Ways of Talking

- Kick your buts
- Pointing at the root of words e.g.:

CONFIDENCE

CON FIDES

COURAGE

COUER AGE

The University of Edinburgh is a charitable body, registered in Scotland, with registration number SC005336

In therapeutic conversations…

- Lots of cues that signal stepping back:

‘Look at what your mind is giving you’

‘Given your history, it’s no surprise that your mind might say that to you’

‘If you could see that thought, what would it look like?’

The University of Edinburgh is a charitable body, registered in Scotland, with registration number SC005336

In therapeutic conversations…

- “I’m having the thought that…”
- “Thank you mind…”
- Having thoughts versus buying thoughts
- Your mind is not always your friend
- Does that thought seem old, familiar?

The University of Edinburgh is a charitable body, registered in Scotland, with registration number SC005336

THE UNIVERSITY *of* EDINBURGH
