# Supplementary material for: A qualitative study of bereavement support volunteers’ views and experiences on an online Acceptance and commitment therapy-based (ACT) training programme
Source: PLoS One. 2025 Dec 8;20(12):e0337321. doi: 10.1371/journal.pone.0337321 (PMC12685200; doi:10.1371/journal.pone.0337321)
Supplement: S3 File — (PDF) [file pone.0337321.s003.pdf]

THE UNIVERSITY of EDINBURGH

Cruse Scotland
Bereavement Support

FUNDED BY
Marie Curie
Care and support through terminal illness

mygrief my way

Support Volunteer Training: Session Three

Dr. David Gillanders

University of Edinburgh

The ACT model: Psychological Flexibility

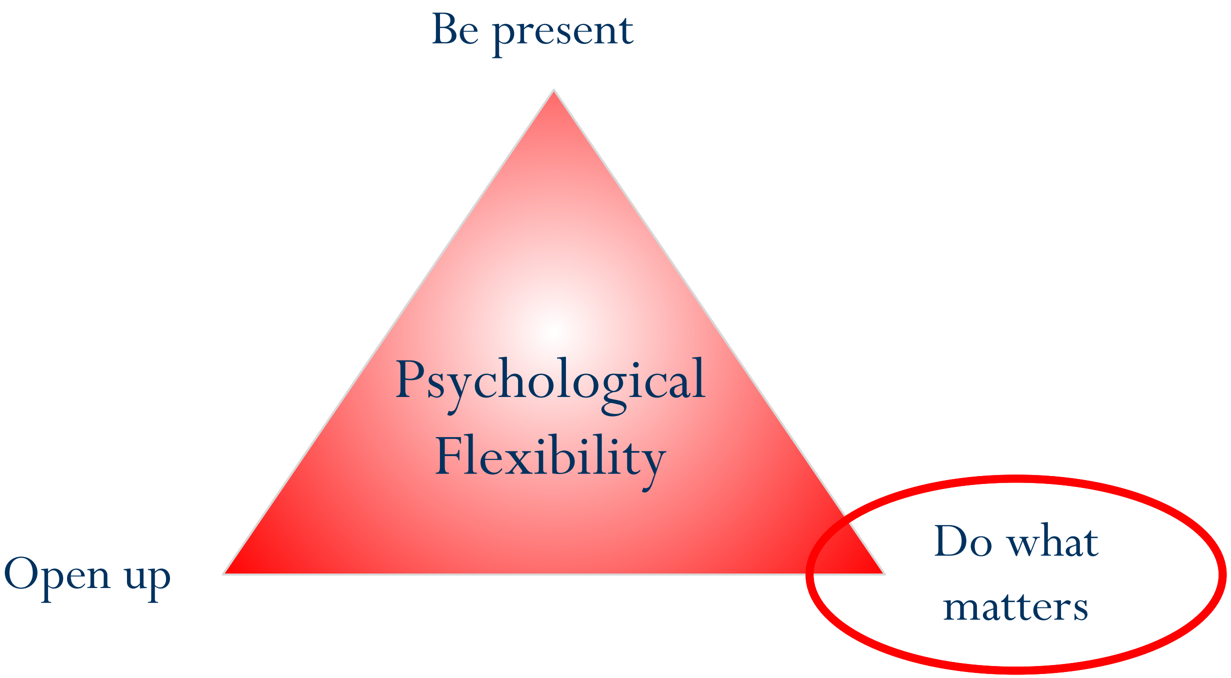

Building engagement

What are values?

"Values are your heart's deepest desires for how you want to behave as a human being. Values are not about what you want to get or achieve; they are about how you want to behave or act on an ongoing basis; how you want to treat yourself, others, the world around you."

Russ Harris, 2010

The Compass Metaphor

- Values as compass directions
- Goals as the towns you pass along the way
- You want both together

The University of Edinburgh is a charitable body, registered in Scotland, with registration number SC005336

Qualities of action

- Being a good parent…..
- What are the qualities you would most want to bring to your parenting, *if you could choose?*
- Choosing sweets metaphor

Time management problems

Steven Covey, 7 Habits of Highly Effective People

Weaving a tapestry

- Think of your life as a tapestry that you have been constantly weaving…

Specific Steps

- Any actions that moves you in the direction of your values
- Overt or covert
- Bold moves or tiny steps
- Particularly actions that you take in the presence of obstacles and barriers

In summary…

Actions → Goals → Values

The Grief Swing

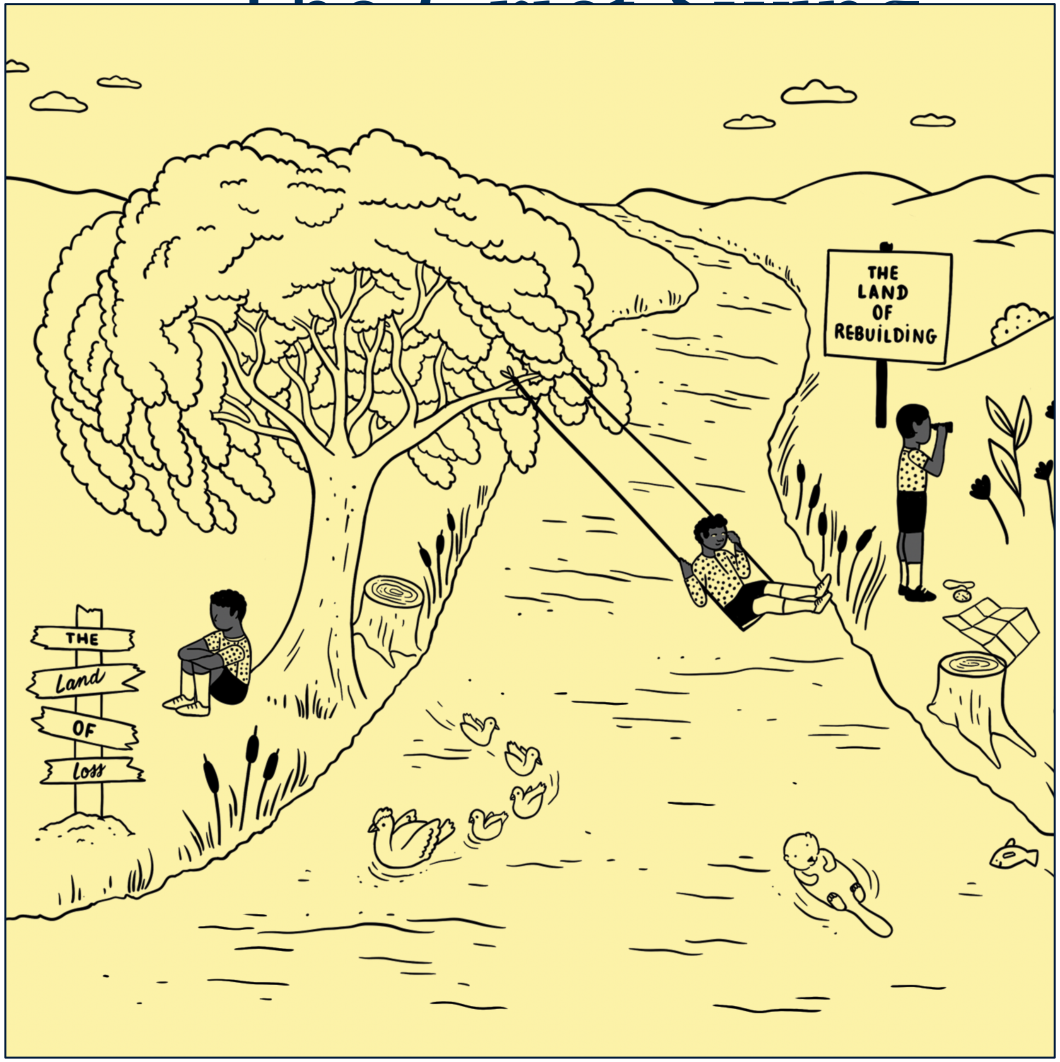

Image credit: Reprinted from the book "You will be OK" by Julie Stokes, Hachette Publishing: www.hachette.co.uk/titles/julie-stokes/you-will-be-okay/9781526363886/ under a CC BY license, with permission from Lauren Boglio: www.boglio.com, 2021.

The work of grief…

- Grief as work or task
- Spending time doing it

- And also….

- Spending time doing other things….

The University of Edinburgh is a charitable body, registered in Scotland, with registration number SC005336

Health, wellbeing, lifestyle

- Supporting yourself to do the work of grieving
- Advice about sleep, nutrition, activity being in nature

Continuing Bonds Rituals

- Little actions that help you carry the memory of your lost person in helpful ways
- That allow you to engage in living, bringing their memory with you
- The memory box

The nursery tree

Your story isn't finished

- When the time is right
- Language around 'moving forward'
- Anticipating obstacles and barriers

THE UNIVERSITY of EDINBURGH

Moving around the tri-flex

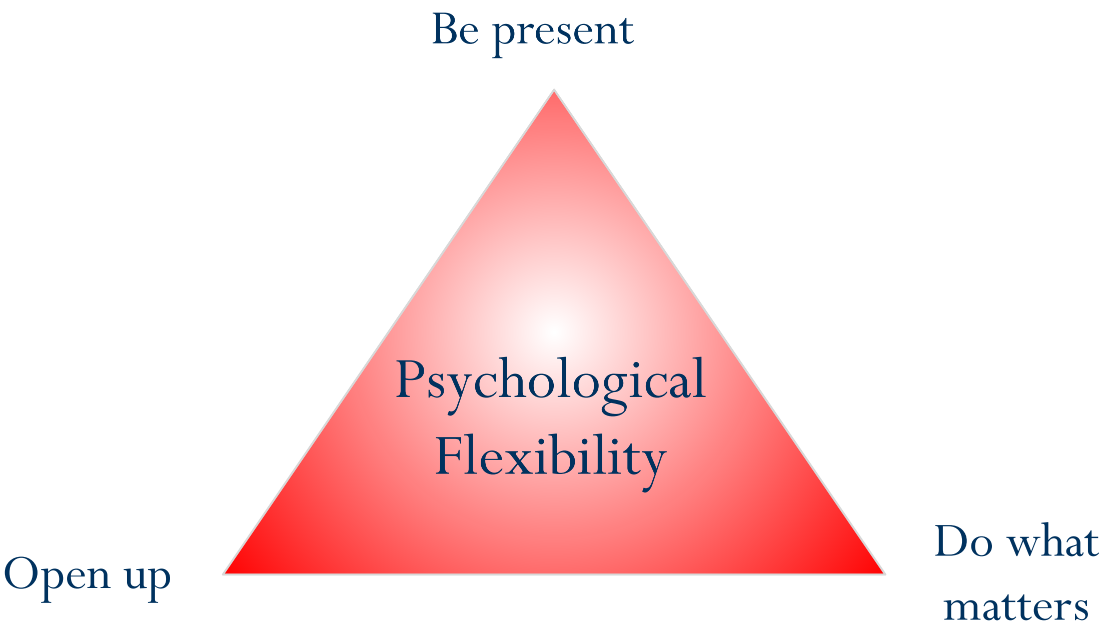

THE UNIVERSITY of EDINBURGH

mygrief my way

Cruse Scotland
Bereavement Support

FUNDED BY
Marie Curie
Care and support through terminal illness
