# Supplementary material for: A qualitative study of bereavement support volunteers’ views and experiences on an online Acceptance and commitment therapy-based (ACT) training programme
Source: PLoS One. 2025 Dec 8;20(12):e0337321. doi: 10.1371/journal.pone.0337321 (PMC12685200; doi:10.1371/journal.pone.0337321)
Supplement: S4 File — (PDF) [file pone.0337321.s004.pdf]

THE UNIVERSITY of EDINBURGH

Cruse Scotland Bereavement Support

FUNDED BY Marie Curie Care and support through terminal illness

mygrief my way

Support Volunteer Training: Session Four

Dr. David Gillanders

University of Edinburgh

The University of Edinburgh is a charitable body, registered in Scotland, with registration number SC005336

THE UNIVERSITY of EDINBURGH

The look and feel of My Grief My Way

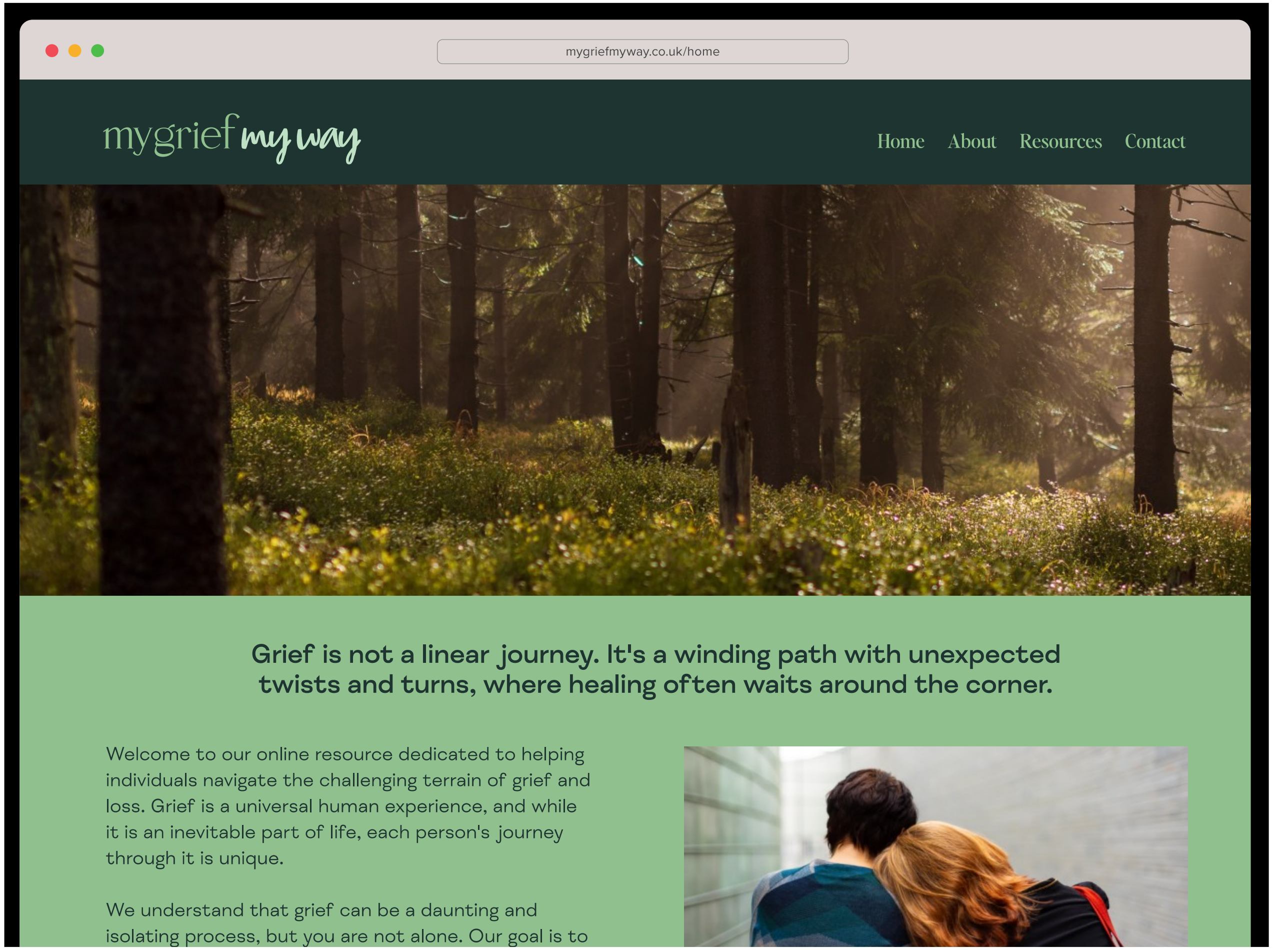

THE UNIVERSITY of EDINBURGH

The site structure

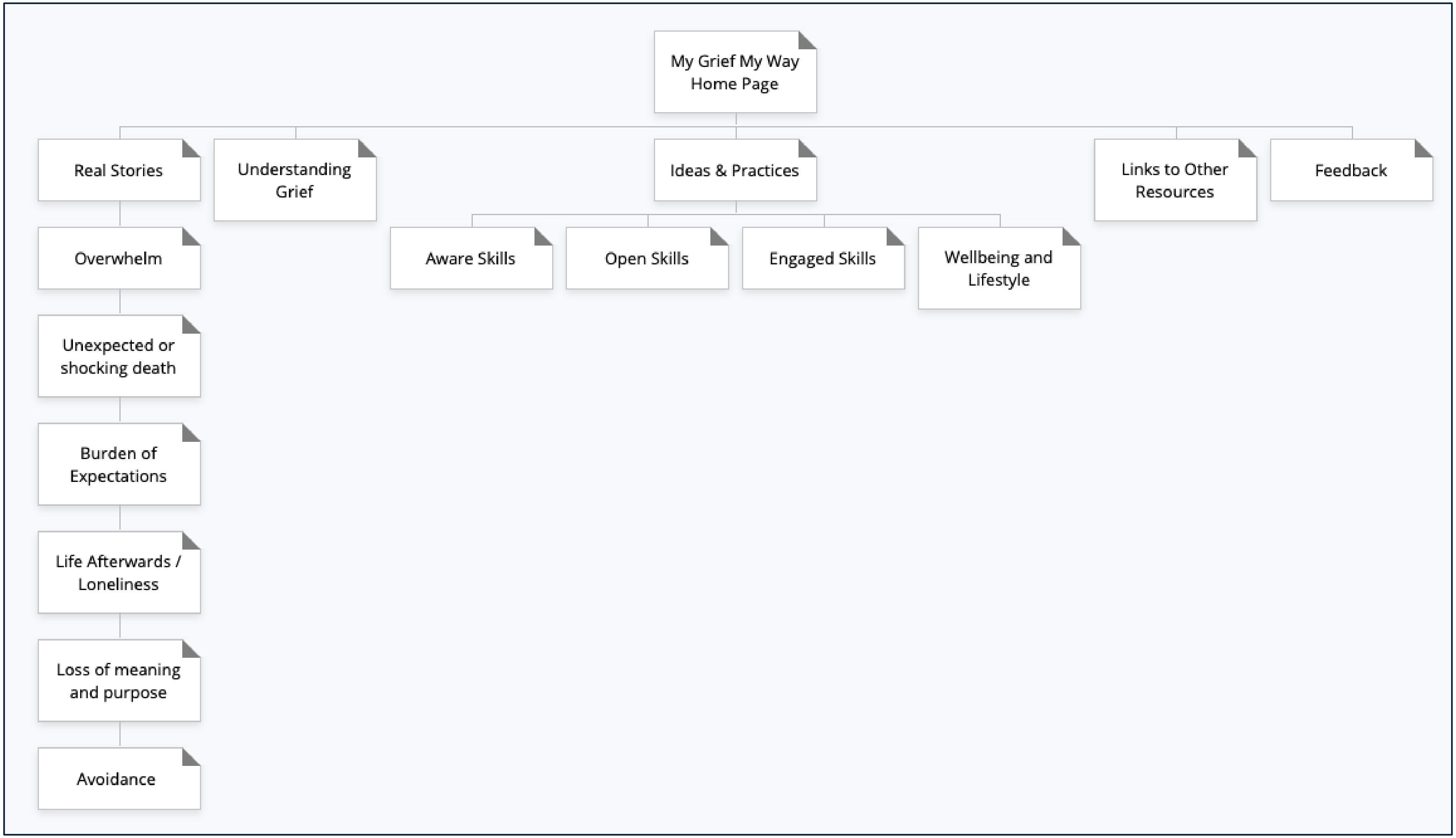

Real Stories

- Purpose is engagement and normalisation
- Brief text introduction to each of the speakers
- Not scripted or directed, some light interview prompts
- Themes drawn from our first 9 months of programme theory development
- People spoke about things that we could relatively easily put together into the themes

Themes

- Overwhelming feelings
- Unexpected Death
- Physical Effects
- Expectations of Self and Others
- Avoidance
- Life After
- What Helped

The University of Edinburgh is a charitable body, registered in Scotland, with registration number SC005336

Example: Physical Effects

The University of Edinburgh is a charitable body, registered in Scotland, with registration number SC005336

www.ed.ac.uk

Understanding Grief

- Brief Text and videos from David

- Themes: Range of different emotions
  Things that can make grief more difficult
  Intrusive thoughts and memories
  The Grief Swing
  How you are responding
  Physical impacts of grief
  Continuing Bonds

- Practical aspects of loss with links to other resources

The University of Edinburgh is a charitable body, registered in Scotland, with registration number SC005336

Example: How are you dealing with grief?

Ideas & Practices

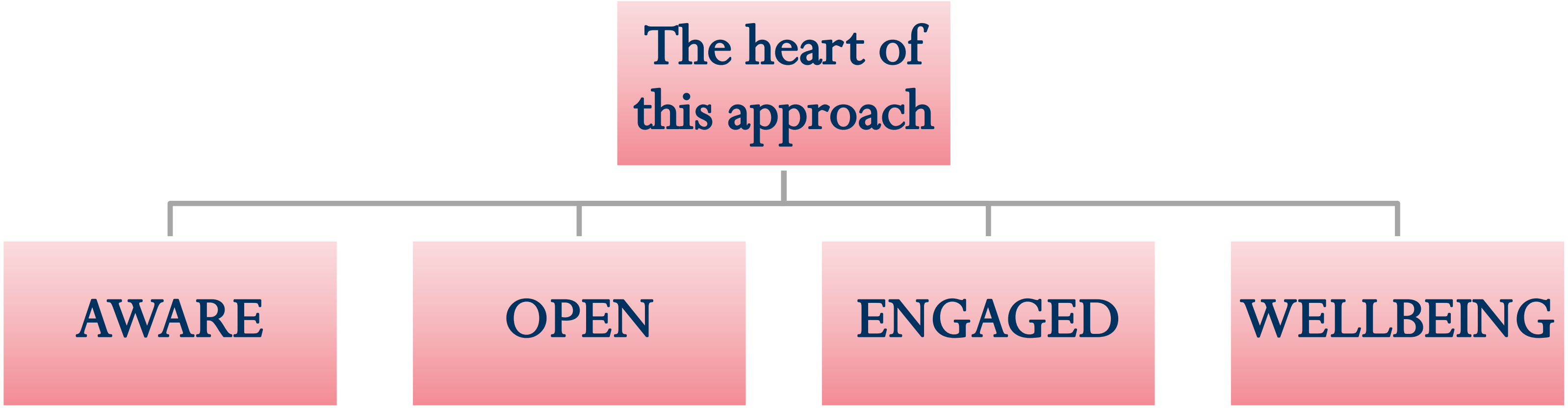

AWARE

- Brief text
- Introductory Video by David
- How are you talking to yourself?
- Holding it together
- Audio exercises: Developing awareness
  Self-soothing
  Dropping Anchor
- Worksheets: How are you dealing with grief?
  What's the emotion?

OPEN

- Brief text
- Introductory Video by David
- The Guest House Poem
- Audio exercises: Leaves on a stream
  Passengers on the bus
- Worksheets: Catching self-talk
- Letter writing exercise

ENGAGED

- Brief Text
- Introductory Video by David
- Poem 'Start Close in' by David Whyte
- Audio Exercises: Imagine one year from now
  What would they want for you?
- Worksheets: Values Compass
  Goals and Actions
  Bull's eye or similar ?
  Dealing with expectations
  Your story isn't finished
  Continuing bonds rituals

THE UNIVERSITY of EDINBURGH

Wellbeing & Lifestyle

- Brief Text
- Video: Treating grief as work, looking after yourself, addressing skills, other barriers
- Sleep advice
- Eating – links to simple recipes / batch cooking
- Being in nature
- Social Connection
- Activity / Gentle exercise

The University of Edinburgh is a charitable body, registered in Scotland, with registration number SC005336

Example: Supporting yourself as you grieve

Other Resources

- Links to other organisations and sites

The University of Edinburgh is a charitable body, registered in Scotland, with registration number SC005336

Specific Questions?

- The ACT videos?

- The worksheets?

Starting from where you are at

An ACT lens on your current practice

- What are the core principles and techniques that you currently use in supporting people?

- Can you group those ideas according to:

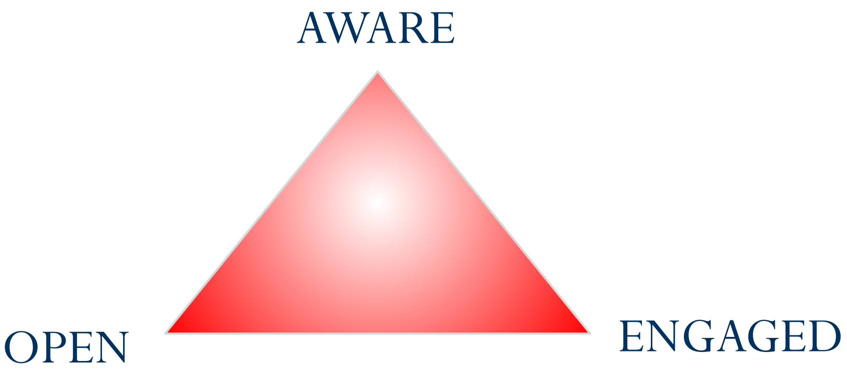

Your future practice

- What ideas have you taken from this training that you might use?
- What barriers arise for you as you think about doing something different?
- What could you use from this work to help you navigate that?

The University of Edinburgh is a charitable body, registered in Scotland, with registration number SC005336

What will it be like?

- Very broad range of ways that you might support people

More or less the same ⟷ Revised and restructured

If in doubt….

- What matters to this client?
- What is the consequence of their actions?
- What am I aware of in the moment now?
- Step in the direction of my own values

Next steps

- Website complete by around the 11th January
- Explore it and give feedback by 17th January
- We start recruiting participants around 17th January
- We want to start delivering to them around 29th January

- Three cohorts of about 15 participants from Feb to July 2024
- Two supervision / consultation groups during each cohort
- Email responses from me in between

The University of Edinburgh is a charitable body, registered in Scotland, with registration number SC005336

Supervision / Consultation

- Experiences – good and bad
- What has worked and not worked
- Barriers / difficulties – internal and external
- Questions
- Concerns
- What you need

Final reflections

THE UNIVERSITY of EDINBURGH

mygrief my way

FUNDED BY

Marie Curie

Care and support through terminal illness

Cruse Scotland

Bereavement Support
