# Supplementary material for: A qualitative study of bereavement support volunteers’ views and experiences on an online Acceptance and commitment therapy-based (ACT) training programme
Source: PLoS One. 2025 Dec 8;20(12):e0337321. doi: 10.1371/journal.pone.0337321 (PMC12685200; doi:10.1371/journal.pone.0337321)
Supplement: S5 File — (DOCX) [file pone.0337321.s005.docx]

**Supporting Material S5.** Topic Guide: Bereavement Support Volunteer.


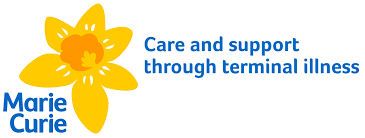

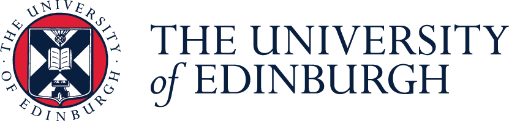


**Development of an online self-directed Acceptance and Commitment Therapy (ACT) intervention to improve ability to cope and quality of life after bereavement**

**Semi-structured Interview/Focus Group – Bereavement Support Volunteer Training and Delivery of ACT**

You recently took part in My Grief My Way study (MGMW). We would like to hear your views and opinions on your participation in supporting the delivery of ACT-based on-line bereavement materials with people affected by grief. We expect the interview/focus group to last between 60 and 90 minutes, but we can stop to take a break at any point, and you can finish the interview at any time without giving an explanation.

[If participant has consented to be recorded, switch on the recorder]

May I double-check that you are happy for this interview to be recorded?

[Continue recording, if participant has confirmed their consent.]

**ACT Training**

1. **Initial Thoughts**
2. What were your reasons for agreeing to take part in ACT training?
3. **Volunteer Experiences**
4. What do you think of the materials used to teach the principles of ACT?
5. How easy was it for you to understand the principles of ACT?
6. How confident were you in delivering the principles of ACT to your clients after training?
7. Will you continue to use ACT in your work? If so, why/how? If not, please let me know.
8. Overall, how did you find the training?
9. **Suggestions/recommendations**

Do you have any suggestions or recommendations that you feel may improve the training?

1. Length of course?
2. Materials used?
3. Online/face-to-face preferences?
4. One-to-one/group?

**Delivery of ACT**

1. **Initial Thoughts**
2. How comfortable/confident were you about using materials to help support bereaved individuals?
   - 1. General principles of ACT.
     2. Online materials.
3. **Bereavement Support Volunteer Experiences**
4. Tell me a little bit about your client’s engagement? For instance, how many sessions did you and your client complete during the six to eight-week provision? How regularly did you and your client use website materials together? Can you talk this through with me (daily, weekly, planned/unscheduled)?
5. How easy (or not) was it for you to access or navigate the website?
6. How easy (or not) was it for you and your client to find materials that were of interest to you/them?
7. How did website materials help you in the delivery of ACT-based principles? If so, how? If not please let me know.
8. How does My Grief My way compare with how you usually provide bereavement support?
9. **Suggestions/recommendations**
   1. How could we improve the resource (MGMW)?
      1. Add or remove from the website to improve your client’s experience?
      2. Or your own experience?
   2. Would you recommend the MGMW website to friends and family? If so, why? If not please let me know.
   3. Would you use the resource in the future to support bereaved clients?
      1. If so, why? If not, please let me know.

Closing question

Is there anything else you would like to discuss that has not already been covered?

Thank you very much for taking part in this interview.
