# Supplementary material for: A qualitative study of bereavement support volunteers’ views and experiences on an online Acceptance and commitment therapy-based (ACT) training programme
Source: PLoS One. 2025 Dec 8;20(12):e0337321. doi: 10.1371/journal.pone.0337321 (PMC12685200; doi:10.1371/journal.pone.0337321)
Supplement: S6 File — (DOCX) [file pone.0337321.s006.docx]

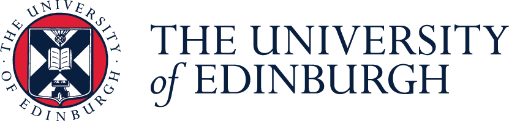

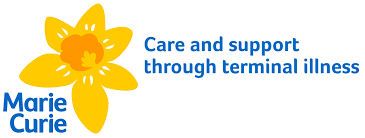


**Development of an online self-directed Acceptance and Commitment Therapy (ACT) intervention to improve ability to cope and quality of life after bereavement**

**Participant Information Sheet – Bereavement Support Volunteer**

You are invited to take part in a research study called My Grief My Way. Dr Anne Finucane, Marie Curie Senior Research Fellow and Dr David Gillanders, Senior Lecturer at the University of Edinburgh are leading this research. Before you decide whether to take part, it is important for you to understand why the research is being carried out and what it will involve. Please take time to read the following information carefully. Talk to others about the study if you wish. Contact us if there is anything that is not clear, or if you would like more information.

**What is the purpose of the study?**

Our study involves designing an online psychological support website to help people cope with grief and to improve quality of life and wellbeing after bereavement. As part of this, we will provide training to bereavement support volunteers at Cruse Scotland and Marie Curie in Acceptance and Commitment Therapy (ACT), so that volunteers providing support to bereaved people using the website are familiar with ACT resources for bereavement support and can draw on ACT to help them provide bereavement support.

**Why have I been invited to take part?**

We are working with Marie Curie and Cruse Scotland to develop this resource. We invited you to take part because you have had bereavement support training and currently provide bereavement support through Marie Curie or Cruse Scotland. Participants must also be able to speak English. You are also considering taking part because you are interested in receiving training in Acceptance and Commitment Therapy.

**Do I have to take part?**

No, it is entirely up to you to decide whether to take part. If you decide to take part, you are still free to withdraw at any time and without giving a reason. Deciding not to take part or withdrawing from the study will not affect volunteer arrangements with Marie Curie or Cruse Scotland bereavement support services. If you decide to withdraw during the study, you will be asked if the research team can use data already collected about you in their findings. You do not have to agree to this.

**What will happen if I take part?**

Please discuss participation in this study with your manager. If you do decide to take part, please keep this Information Sheet. You will be sent a link to an online Informed Consent Form for you to complete to show that you understand your rights in relation to the research, and that you are happy to participate.

You will also be asked to complete a brief online questionnaire, which will give us some information about your current circumstances (e.g., age, gender, ethnicity, length of time providing bereavement support etc). This will take around 3 minutes to complete. The information collected will only be used to describe the volunteers involved. Data will be stored in an encrypted file in the secure University of Edinburgh data store.

You will be invited to four half-day training sessions covering the principles of Acceptance and Commitment Therapy. These sessions will take place online over a period of about 4 weeks. On completion of the training, your manager will be informed that you are ready to offer bereavement support to a research participant. The co-ordinator at your organisation (Cruse Scotland or Marie Curie) will match you to a participating bereaved individual, as would usually be the case. The only difference will be that you will offer support to people who are accessing the My Grief My Way website, and you can use your newly acquired ACT skills and exercises when supporting them, in conjunction with your usual approach.

You will be given a link to our website so that you can help guide your bereaved person through website materials by holding a 45-50 minute telephone conversation each week or fortnight for up to six sessions. This meeting will be arranged by your organisation in line with the current procedures for doing this. During this time, you will be supported by the research team, including our lead Clinical Psychologist, David Gillanders, who will arrange group supervision opportunities to volunteers who are new to supporting bereaved individuals using Acceptance and Commitment Therapy. The website site will be live so that anonymised site usage data can be collected using Google Analytics and will inform intervention development.

Following the training session, and after you have had the chance to support a bereaved individual, you will be asked for your views and experiences of delivering the intervention to find out what works, what does not so that materials are as useful and valuable as possible. We will obtain your views and experiences through online focus groups or an individual online semi-structured interview (depending on participant preference). Interviews will last up to one hour and the focus group will last up to 1.5 hours.

With your consent, the focus groups/semi-structured interviews will be audio recorded so that we have an accurate account of your experiences. Information you provide will be transcribed and anonymised within two-three weeks of occurring so that you will not be identifiable. The audio recording will be erased as soon as transcription is completed and checked by a member of the research team, within a further two-three weeks.

**What are the possible advantages and benefits of taking part?**

You will receive training in Acceptance and Commitment Therapy, which we hope you find enjoyable and useful, and will likely enhance your skills in providing bereavement support. Your views will also help shape the development of online bereavement support resources, which will hopefully be accessed by many bereaved individuals in the future.

**What are the possible disadvantages and risks of taking part?**

The main disadvantage is the time involved in attending the training. Furthermore, even though you are currently providing bereavement support in your volunteer role, it is possible therefore, that you may feel emotional or upset when supporting bereaved individuals taking part in this study. Please be assured that you will be supported by your organisation - Marie Curie or Cruse Scotland, should you require additional help. The research team will also be on hand to direct you to additional support and resources if needed.

**Will my participation in the study be kept confidential?**

All the information we collect during the course of the research will be kept confidential and there are strict laws that safeguard your privacy at every stage. We will not share any information about you with any other organisation except if you tell us about a serious risk of harm to yourself or others, in which case we may need to disclose to relevant organisations in order to manage that risk and fulfil our duty of care.

**How will we use information about you?**

We will need to use information from you for this research project.  This information will include your:

- - - Name
    - Contact details
    - Organisation you volunteer for
    - Gender
    - Ethnicity
    - Length of time as a volunteer
    - Skill level

People will use this information to do the research or to check your records to make sure that the research is being done properly.

People who do not need to know who you are will not be able to see your name or contact details. Your data will have a code number instead.

We will keep all identifiable information about you safe and secure for a period of one year.

If you consent to being audio recorded, all recordings will be destroyed once they have been transcribed. Your data will only be viewed by the researcher/research team. All electronic data will be stored on a password-protected computer file and all paper records will be stored in a locked filing cabinet. Your consent information will be kept separately from your responses in order to minimise risk.

Once we have finished the study, we will keep some of the data so we can check the results. We will write our reports in a way that no-one can work out that you took part in the study.

### What are your choices about how your information is used?

- You can stop being part of the study at any time, without giving a reason, but we will keep information about you that we already have.
- We need to manage your records in specific ways for the research to be reliable. This means that we won’t be able to let you see or change the data we hold about you.

### Where can you find out more about how your information is used?

You can find out more about how we use your information

- At http://www.ed.ac.uk/records-management/privacy-notice-research
- by asking one of the research team
- by sending an email to the University of Edinburgh Data Protection Officer at [dpo@ed.ac.uk](mailto:dpo@ed.ac.uk)

The University of Edinburgh is the sponsor for this study based in the United Kingdom. We will be using information from you in order to undertake this study and will act as the data controller for this study. This means that we are responsible for looking after your information and using it properly. The University of Edinburgh will keep identifiable information about you for 1 year after the study has finished and your anonymised data for a minimum of 3 years.

**What will happen to the results of the research study?**

Results of the study will be disseminated to Marie Curie and Cruse Scotland bereavement support services. Findings will also be presented at national and international conferences. The study will be written up for publication in peer-reviewed, open access journals. You will not be identifiable from any of these works. You can request a copy of the results from the research team if you wish by contacting core team members at the end of this information sheet. This summary will be sent to participants by email.

**Who is organising and funding the study?**

This study has been organised by Drs Anne Finucane and David Gillanders from the School of Health in Social Science at the University of Edinburgh who will co-lead the research team. Dr Anne Canny is Research Associate, leading the research on a day-to-day basis. The research is sponsored by the University of Edinburgh.

This study has been funded by Marie Curie (www.mariecurie.org.uk/).

**Who has reviewed the study?**

The study proposal has been reviewed by the Clinical Psychology Ethics Committee at the School of Health in Social Science, University of Edinburgh.

**What happens now?**

You do not have to do anything. If you have given Cruse Scotland or Marie Curie permission to share your contact information with the research team, a team member (Anne Canny) will be in touch with you to answer any questions you may have before you decide whether you want to take part. If you decide to take part, you will be asked to complete a consent form and a background questionnaire. We will then provide you with some possible dates for training in Acceptance and Commitment Therapy and the My Grief My Way resource.

**What if there are any problems?**

If you have concerns about any aspect of the study or experience any problems, please do not hesitate to contact your bereavement organisation or core members of the research team listed at the end of this information sheet.

**If you have any further questions about the study, please contact:**

Anne Canny: [anne.canny@ed.ac.uk](mailto:anne.canny@ed.ac.uk)

Anne Finucane: [a.finucane@ed.ac.uk](mailto:a.finucane@ed.ac.uk)

David Gillanders: [david.gillanders@ed.ac.uk](mailto:david.gillanders@ed.ac.uk)

**Study telephone number:** 07752229606

If you would like to discuss this study with someone independent of the study, please contact: School of Health in Social Science Director of Research, Dr Angus MacBeth, Angus.Macbeth@ed.ac.uk

**Complaints**

If you wish to make a complaint about the study, please contact: Prof Matthias Schwannauer, Head of the School of Health in Social Science: headofschool.health@ed.ac.uk

**Thank you for taking time to read this information sheet**
