# Supplementary material for: A qualitative study of bereavement support volunteers’ views and experiences on an online Acceptance and commitment therapy-based (ACT) training programme
Source: PLoS One. 2025 Dec 8;20(12):e0337321. doi: 10.1371/journal.pone.0337321 (PMC12685200; doi:10.1371/journal.pone.0337321)
Supplement: S7 File — (PPTX) [file pone.0337321.s007.pptx]

## Slide 1
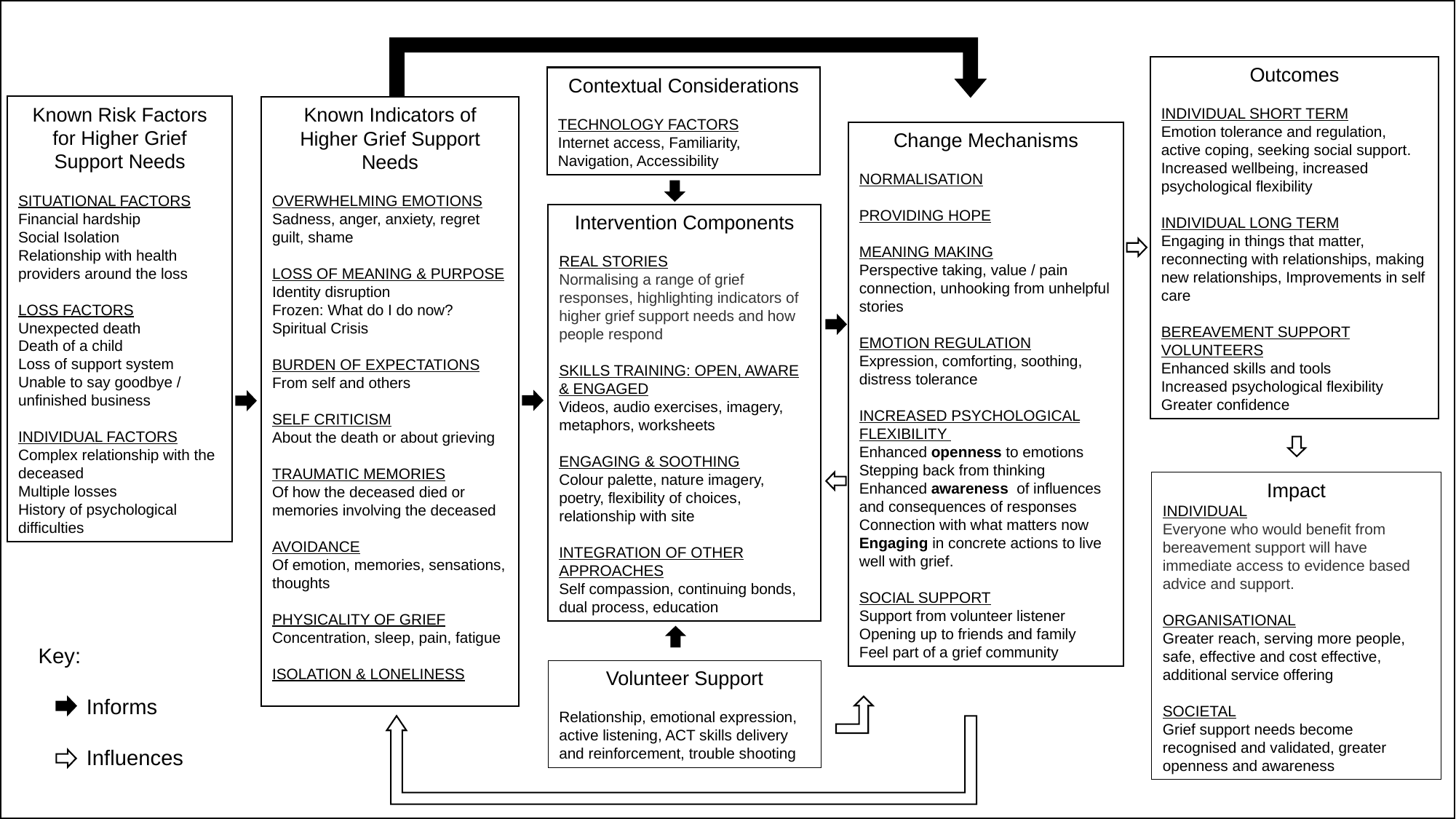

Outcomes
INDIVIDUAL SHORT TERM
Emotion tolerance and regulation, active coping, seeking social support. Increased wellbeing, increased psychological flexibility
INDIVIDUAL LONG TERM
Engaging in things that matter, reconnecting with relationships, making new relationships, Improvements in self care
BEREAVEMENT SUPPORT VOLUNTEERS
Enhanced skills and tools
Increased psychological flexibility
Greater confidence
Contextual Considerations
TECHNOLOGY FACTORS
Internet access, Familiarity, Navigation, Accessibility
Known Risk Factors for Higher Grief Support Needs
SITUATIONAL FACTORS
Financial hardship
Social Isolation
Relationship with health providers around the loss
LOSS FACTORS
Unexpected death
Death of a child
Loss of support system
Unable to say goodbye / unfinished business
INDIVIDUAL FACTORS
Complex relationship with the deceased
Multiple losses
History of psychological difficulties
Known Indicators of Higher Grief Support Needs
Overwhelming emotions
Sadness, anger, anxiety, regret guilt, shame
Loss of meaning & purpose
Identity disruption
Frozen: What do I do now?
Spiritual Crisis
Burden of expectations
From self and others
SELF CRITICISM
About the death or about grieving
TRAUMATIC MEMORIES
Of how the deceased died or memories involving the deceased
AVOIDANCE
Of emotion, memories, sensations, thoughts
PHYSICALITY OF GRIEF
Concentration, sleep, pain, fatigue
ISOLATION & LONELINESS
Change Mechanisms
NORMALISATION
PROVIDING HOPE
MEANING MAKING
Perspective taking, value / pain connection, unhooking from unhelpful stories
EMOTION REGULATION
Expression, comforting, soothing, distress tolerance
INCREASED PSYCHOLOGICAL FLEXIBILITY
Enhanced openness to emotions
Stepping back from thinking
Enhanced awareness of influences and consequences of responses
Connection with what matters now
Engaging in concrete actions to live well with grief.
SOCIAL SUPPORT
Support from volunteer listener
Opening up to friends and family
Feel part of a grief community
Intervention Components
REAL STORIES
Normalising a range of grief responses, highlighting indicators of higher grief support needs and how people respond
SKILLS TRAINING: OPEN, AWARE & ENGAGED
Videos, audio exercises, imagery, metaphors, worksheets
ENGAGING & SOOTHING
Colour palette, nature imagery, poetry, flexibility of choices, relationship with site
INTEGRATION OF OTHER APPROACHES
Self compassion, continuing bonds, dual process, education
Impact
INDIVIDUAL
Everyone who would benefit from bereavement support will have immediate access to evidence based advice and support.
ORGANISATIONAL
Greater reach, serving more people, safe, effective and cost effective, additional service offering
SOCIETAL
Grief support needs become recognised and validated, greater openness and awareness
Key:
 Informs
 Influences
Volunteer Support
Relationship, emotional expression, active listening, ACT skills delivery and reinforcement, trouble shooting
