# Supplementary material for: A qualitative study of bereavement support volunteers’ views and experiences on an online Acceptance and commitment therapy-based (ACT) training programme
Source: PLoS One. 2025 Dec 8;20(12):e0337321. doi: 10.1371/journal.pone.0337321 (PMC12685200; doi:10.1371/journal.pone.0337321)
Supplement: S8 File — (in preparation). (DOCX) [file pone.0337321.s008.docx]

**A logic model to guide “My Grief My Way”: An intervention development study for a digital psychological support package for unmet bereavement support needs.**

**Abstract**

**Background**: Logic models are simplified graphics that guide the development and evaluation of complex interventions. This paper describes a logic model for an online intervention to improve coping and quality of life after bereavement.

**Methods**: A combination approach to intervention development was used. Evidence was synthesised in iterative cycles from: (i) research literature; (ii) interviews with therapists; (iii) workshops with bereaved people; (iv) workshops with bereavement support professionals; and (v) expertise of the research team.

**Results**: The logic model illustrated the links between: risk factors for and indicators of grief support needs, contextual considerations for online interventions, intervention components, change mechanisms, short and long term intended outcomes at the individual and organisational level, as well as broader impacts.

**Conclusions**: The logic model guided the intervention development process, fostering collaboration and synthesis of multiple sources. The description of the process will be useful to other intervention developers.

**Plain Language Summary**

**Using a framework to guide the creation of a website to help people cope after bereavement.**

Grief after bereavement can be hard to handle. Most people manage with family and friends for support, but about 1 in 3 people need additional support. Providing bereavement support is complicated and resource intensive. To offer additional help our team created a new website called “My Grief My Way”. This paper describes the process that we used to create a logic model to guide the development of the website. A logic model is a graphic that captures important elements and features of an intervention. The logic model spells out what the problem is, what the elements of the intervention are, why they have been chosen and what outcomes we hope to achieve. This paper shows how the logic model provided a clear framework that successfully brought together our expertise, the experiences of bereaved people, the research evidence, and theories of grieving. Our purpose is to offer guidance to other intervention developers by showing how useful the logic model was in guiding our work, whilst also making explicit the theory and guiding principles that underpin our intervention.

**Keywords**: Logic Model, Intervention Development, Bereavement, Grief, Online, Digital.

**Introduction**

The evaluation of psychological interventions via clinical trials is complex, time consuming and expensive. It is therefore crucial that interventions are developed based on robust evidence and theory, and are ideally known to feasible and acceptable prior to evaluation (Skivington et al., 2021). A taxonomy of eight approaches to intervention development has been described by O’Cathain and colleagues, to help treatment development teams to conceptualise their approach (O’Cathain et al., 2019). These approaches are illustrated in Table 1.

[Insert Table 1 HERE]

Given the complexity of concepts and processes involved in an intervention development study, and the strong emphasis in a number of these approaches on engaging meaningfully with people with lived experience (i.e., patient and public involvement (PPI)), research teams need methods that can guide their development study and communicate this complexity in ways that are accessible to people without technical expertise. Logic models represent such a method.

A logic model is a graphical representation of the components of an intervention (Greene, 2018). The exact format of a logic model is flexible, but they typically contain descriptors of the context of an intervention, the particular targets that the intervention intends to influence, the mechanisms or processes by which those targets will change, moderators or facilitators of change, and outcomes or impacts in both the short and long term. Logic models represent an important step in making explicit the foundations and assumptions of an intervention. This promotes transparency and is fundamental to a high-quality future evaluation of an intervention. Iterative refinement of the logic model during piloting is often a stage prior to full evaluation. Logic models also aid communication with experts by experience. Well-informed experts by experience can more meaningfully engage and effectively shape intervention development. This paper describes the development of a logic model to guide an intervention development study around unmet bereavement support need.

The context for this intervention development study was an awareness of the significant unmet needs of a substantial proportion of people who are bereaved. Whilst approximately 60% of people grieve and cope effectively with only support from family and friends, around 30% experience ‘moderate’ levels of difficulties with grief, and can benefit from opportunities to discuss and reflect upon their grief with other bereaved people or with volunteer or professional counsellors (Aoun et al. 2015; Harrop et al. 2020a). Around 10% experience significant challenges to their mental health and social or occupational functioning (Aoun et al., 2015; Lundorff et al., 2017). The COVID-19 pandemic led to an estimated 6.8 million bereavements in the UK in 2020-2021, an additional 750,000 compared to the yearly average from 2015-2019 (UK Commission on Bereavement, 2022). The circumstances of these bereavements also contained many features likely to be associated with higher grief support needs. For example, many people died unexpectedly, many bereaved people were not able to say goodbye to the deceased, could not attend funerals, or could not visit friends or family for contact and social support, with devastating consequences for their grief and coping (Torrens-Burton et al. 2022). Reflecting these difficult circumstances, higher levels of prolonged grief disorder and other psychological conditions have been reported among people bereaved at this time (Harrop et al., 2023, Breen et al., 2022; Neimeyer & Lee 2022; Eisma & Tamminga, 2022). Public and third sector services that provide bereavement support in the UK have unsurprisingly struggled to meet the increased need (UK Commission on Bereavement, 2022).

In this context, we developed an intervention that would address the unmet bereavement support needs of the 30% of people considered to have moderate support needs. We wanted the intervention to be accessible, and based on strong theory and robust evidence. We chose Acceptance and Commitment Therapy (ACT: Hayes, Strosahl & Wilson, 2012) as the underpinning theory of psychological change based on its strong empirical and theoretical basis across a wide range of settings and problems (e.g. Gloster et al., 2020). We also wanted the intervention to be person-based, co-produced with people who have lived experience of grief, as well as service providers and academic and practice experts in this field. Finally, we wanted the intervention to be easy to implement into routine practice settings, and to address need at level 1 and 2 of the NICE Bereavement Support Framework (NICE, 2004). Given the complexity of inputs, processes and outputs, and the desire for a high degree of expert-by-experience engagement, a logic model was developed to guide and communicate the process.

**Methods**

*Development Approach*

Based upon the taxonomy of approaches to intervention development described by O’Cathain et al. (2019), a combination approach was taken. Core values that underpinned this work were that the intervention should be co-produced with experts-by-experience and contain significant elements of person-based and user-centred intervention development. In addition, the intervention incorporated empirical evidence and associated psychological theory. Finally, we wanted the resultant intervention to be easily implementable, and to address the issue of scarcity of access to specialist psychological practitioners. The logic model integrated these approaches.

Five sources were drawn on to create the logic model, as illustrated in Figure 1 and described in the text below.

[Insert Figure 1 HERE]

*Figure 1: Sources used to create the logic model*

*Engagement with People with Lived Experience of Bereavement*

Patient and Public Involvement (PPI) was central to this study from the beginning. The research team advertised the opportunity for involvement with the study within organisations that provide bereavement support. Nine people with experience of grief self-referred and joined the study PPI group. This group had diversity of experience in terms of relationship to the deceased, expected versus unexpected death, as well as gender and ethnicity. This group met using MS Teams, on four occasions between June 2023 and January 2024, and responded to email requests. They reviewed and contributed to all concepts and ideas, intervention materials, video and audio media, as well as all evaluation methods and materials. They were paid for their time. The Public Involvement in Research Impact Toolkit (PIRIT) (Cardiff University, n.d.) was used to document their contribution to the process of building both the logic model and the intervention and evaluation.

*Engagement with Organisations and People Delivering Bereavement Support*

The research team included people who provide bereavement support services as collaborators. This included bereavement team leads and service providers in national charities. In addition, the research team undertook a qualitative study of nine practitioners’ experiences of using Acceptance and Commitment Therapy with people who are struggling with grief. This study was published separately (citation removed for blinding). The findings of this study were shared with stakeholders and informed the development of the logic model.

*Engagement with Theories of Grief and Evidence on Bereavement Support*

Members of the research team brought expertise relating to grief and bereavement support studies, well-established theories of grieving (e.g., Dual Process Theory (Stroebe & Schut, 1999); Continuing Bonds Theory (Klass & Steffen, 2018); Meaning Making (Neimeyer et al., 2010)) and philosophical perspectives on grief (e.g., Cholbi, 2021). Research data relating to thwarted or unsuccessful grieving, including controversies around prolonged grief disorder as a diagnostic category were also part of these conversations (e.g., Prigerson et al., 2024).

The bereavement support needs and outcomes initially considered for the intervention - ability to cope, quality of life, and wellbeing - were directly informed by previous research by members of the team (citation removed for blinding; citation removed for blinding). The intervention was also located within the Public Health Model for Bereavement Support and NICE three component model, as a component 1 and 2 intervention for those experiencing low to moderate levels of grief, as opposed to prolonged grief disorder that would require more intensive specialist or psychological intervention (Aoun et al., 2015; NICE 2004).

*Engagement with Theories of Psychological Intervention*

The lead author is an international expert and researcher in the field of Acceptance and Commitment Therapy or Training (ACT), especially as applied to living with chronic illness, cancer, palliative care, care giving and health professional wellbeing. It was therefore natural that ACT would be likely to influence the research team’s thinking about theories of psychological therapy and behaviour change. In addition, our team completed a scoping review of the literature for ACT in palliative care settings, which included ACT for bereavement (citation removed for blinding). These theoretical concepts were shared with all stakeholders and the reflections and discussions shaped the developing logic model. In addition, a conceptual synthesis paper from a theoretical and practitioner perspective was produced from this work (citation removed for blinding).

*Engagement with Evidence for Digital Interventions*

The research team undertook a rapid review of the literature on using digital methods to support people after bereavement. This was published separately (citation removed for blinding). Findings were shared with all stakeholder groups and informed the development of the logic model.

Each of these sources of information and perspectives were shared with the different stakeholders via online and in person meetings, emails, readings, metaphors and articles. The research team’s understanding of context, the processes of change, and effective as well as thwarted grieving, was shaped in iterative cycles of interaction with all of these sources. The research team conducted the above activities between August 2022 and September 2023, recording MS Teams meetings, creating notes, using the Whiteboard function to capture shared notes and completing the PIRIT tool.

An early draft of the logic model (Version 1) was shared with all stakeholder groups in September 2023, discussed and refined between September and December 2023 (Version 2), leading to the creation of the My Grief My Way prototype intervention. This online intervention was user tested in a mixed methods evaluation from February 2024 to September 2024, in two iterative cycles. The logic model was further updated during this evaluation window (Version 3). Results for the evaluation of the intervention development study will be reported separately (citation removed for blinding.). The experience and outcomes of the intervention evaluation were influential in further refining the logic model, which was again shared with all stakeholders in October – December 2024. The finalised version of the logic model (Version 4) is presented in Figure 2, below.

**Results**

The synthesis of all of these sources of information in iterative cycles resulted in the logic model presented in Figure 2 and described in the text.

[Insert Figure 2 HERE]

Figure 2: My Grief My Way Logic Model

*Risk Factors for Higher Grief Support Needs*

Arising principally from expert knowledge of the research evidence in this field, and incorporating lived experience validation, a number of factors known to be associated with a likelihood of higher grief support needs were articulated. These were pragmatically organised under three overlapping categories – situational factors, loss factors and individual factors.

Situational factors included financial hardship and social isolation (e.g., Albuquerque et al., 2024; Harrop et al., 2023). In addition, the bereaved person’s perception of the adequacy of end-of-life care (e.g., was pain and distress adequately controlled) is known to significantly influence the process of grieving. Where care was perceived as inadequate, this was associated with more difficulties grieving (Harrop et al. 2016; 2023; Lobb et al. 2010). A range of loss related factors have also been found to be associated with higher grief support needs, such as an unexpected death, or losses that do not fit the ‘natural order’ such as the death of a child (Sanders, 1980). When loss resulted in a diminished support system, such as the loss of a confidante, this was also seen to be associated with higher grief support needs (Aoun et al., 2015, Harrop et al., 2023. Lobb et al., 2010). Being unable to say goodbye to the deceased, unable to be with them at the end of their life, or any significant unresolved relational issues (unfinished business) were also agreed to be associated with higher support needs.

A number of individual factors were also agreed to be associated with higher grief support needs. These included experiencing multiple losses, the bereaved having a history of psychological difficulties (Lobb et al. 2010), and circumstances where the relationship with the deceased was complex, or unresolved. Such circumstances included abandonment, neglectful, conflictual or abusive relationships.

It is recognised that a number of these suggested risk factors are overlapping and interdependent, for example being prevented from being present at the time of death may have an impact on the bereaved person’s perception of care provided. Such circumstances may fuel both anger and guilt, which may also be sensitised emotions due to a complex or unresolved relationship with the deceased. We considered these risk factors to be additive, with greater number and complexity of factors leading to greater likelihood of higher grief support needs. The arrow illustrates that these risk factors are associated with indicators of higher grief support.

*Indicators of Higher Grief Support Needs*

Integrating expert understanding of psychological theory (e.g., behavioural theory / psychological flexibility), theories of grieving and adaptation (e.g., meaning making, dual process theory) and lived experience perspectives, Indicators of Higher Grief Support Needs illustrated presentation factors that would be likely to indicate having greater difficulty with grief. These factors differ from the ‘Risk Factors’ in that they are more proximal than historic.

Overwhelming negative emotions and difficulty tolerating them was considered to be commonly presented in people with higher grief support needs (Harrop et al., 2021). Overwhelming emotions such as these could also be associated with traumatic memories. These memories were either of the circumstances of the deceased’s death, or of memories involving the deceased, such as abusive experiences. Associated with both overwhelming emotions and traumatic memories were different forms of avoidance. A strong and consistent perspective from grief experts, psychological theory experts and experts by experience was that when people engage in avoidance behaviours (e.g., suppressing memories, thoughts and feelings, not talking to people, substance misuse) grief support needs remain high for a longer period of time. Isolation and loneliness might result partly from avoidance, or as a result of multiple losses leading to a reduced social network. Whilst many people will experience existential concerns following loss, there was a consensus that where this appeared frozen, or deeply disruptive to identity or purpose, that this was associated with higher grief support need (Neimeyer et al., 2010). Where a loss evokes a spiritual crisis or a questioning of faith, this was also seen as an indicator of higher grief support needs.

People who are bereaved will often appraise their own grief responses and may hold beliefs about how they ‘should’ be grieving (Poxon, 2023). These expectations, and associated self-criticism, were felt to also indicate difficulties with grieving. As well as self-expectations, many experts by experiences spoke of how other people communicated their expectations of how they felt the bereaved person should be doing. These were sometimes subtle suggestions, for example with language such as ‘moving on’ or ‘letting go’. Sometimes these communications from others were blatant and critical, such as ‘You should be over this by now’. These expectations or ‘shoulds’ were universally felt to be a burden and were not deemed to facilitate effective grieving. Instead, they would tend to fuel anger towards other people, withdrawal, anger towards the self or guilt and shame and associated self-criticism.

The last theme in this element of the logic model referred to physical impacts of grief, such as symptoms of minor illnesses (recurring colds etc), as well as concentration or memory problems, fatigue, difficulties with sleeping and so on. Our PPI participants spoke about these aspects of their grief being unexpected and harder to make sense of, and we therefore included them as a significant indicator of unmet need.

The arrows that flow from this box in the logic model go to the change mechanisms and to the intervention components, to illustrate how the intervention components have been directly selected to influence these indicators via the change mechanisms, as well as the components being well informed by these indicators directly.

*Contextual Considerations*

Several contextual considerations arose from the rapid review of the literature for online bereavement support (citation removed for blinding) and expert knowledge of other digital interventions. This included awareness of digital poverty, differing levels of familiarity with computers, the internet, and online interventions. These features were critical in informing prototype development and ensuring that accessibility standards in the industry were adhered to (e.g., WCGA 2.2 AA, Crown Copyright 2024). The ability for the intervention to be accessible via a range of devices, including tablets and smartphones (both IoS and Android) was also deemed to be important. Maximising the clarity of navigation was also included in response to user feedback during the intervention evaluation. In addition, levels of literacy were also considered, with language made as simple and clear as possible. Extensive use of images, video and audio presentation of content also helped to make the website accessible for people with lower literacy.

*Intervention Components*

This aspect of the logic model articulated the different features of the online intervention, with awareness of how they would maximise engagement and retention, address indicators of higher grief support needs, and how they would operationalise the main change mechanisms, as illustrated by the arrows flowing into and out from this box in the logic model.

Real stories referred to people with lived experience of bereavement agreeing to tell their story on camera. These were felt to be engaging, and to facilitate normalisation of a very wide range of grief responses. Including diversity of gender, ethnicity, age, and types of loss was also considered important. In addition, we did not script the participants, we asked them to tell us about their experience of bereavement. It was a testament to the near universality of factors seen in ‘Indicators of Higher Grief Support Needs’ that the volunteers gave many examples of these factors. The research team provided the production company with these factors, enabling them to edit the different speakers into short films, organised by theme: “Overwhelming emotions”, “How we block out the pain (Avoidance)”, “Unexpected or Traumatic Deaths”, “Loss of Meaning or Purpose”, “Burden of Expectations”, and “Physicality of Grief”. These real stories allowed the intervention to illustrate the indicators in a compelling, engaging and normalising way.

‘Skills training’ refers to the main theoretical orientation of My Grief My Way. My Grief My Way is based on a modern approach to Cognitive Behavioural Therapy called Acceptance and Commitment Therapy, or ACT (Said as one word, rather than three letters) (Hayes, Strosahl & Wilson, 2012). When used with clients who are not experiencing a mental disorder (for example in workplace stress management), the acronym refers to Acceptance and Commitment Training. This was felt to be important given that our research team did not conceptualise grief as a mental disorder. Throughout the intervention development we were careful to describe My Grief My Way as a support package for people with higher grief support needs, and not as a therapy for people with a disorder (e.g., Prolonged Grief Disorder).

A full description of ACT is beyond the scope of this paper, interested readers are referred to (citation removed for blinding) and (citation removed for blinding) for a detailed description of why ACT can be considered an especially suitable form of intervention for people with higher grief support needs. Briefly, ACT teaches skills that help people to accept their own thoughts, feelings and memories without needless defence, and to take specific actions that are in line with personally held values. The ability to do this is known as ‘Psychological Flexibility’ (Hayes et al., 2006).

The logic model spells out that the intervention should actively train participants in the skills of psychological flexibility. These are described as cultivating ‘AWARENESS’ (noticing what is influencing our behaviour and tracking the consequences of how we respond to challenging situations, including unpleasant emotions, sensations, memories and thoughts). The second aspect of psychological flexibility is developing an “OPEN” response style (stepping back from thinking, reducing the influence of thoughts and beliefs on our behaviour, cultivating an accepting or willing stance towards challenging experiences, including unpleasant emotions, sensations, memories and thoughts). The final aspect of the skills training was the development of an ‘ENGAGED’ response style. This refers to being clear about what is most important to us and making specific actions that lead in the direction of these values. A wide variety of media were used to train psychological flexibility skills. Videos by the first author were used to introduce the concepts, using metaphors, quotes, stories, images, and short animations. These videos were supported by worksheets, audio recordings, diaries, and monitoring forms.

A further important component of the intervention was the overall aesthetic, and relationship that the user would develop with the site. Given the importance of consistency, trust and a sense of psychological safety in any kind of support setting, features such as having the same presenter, who was an expert in ACT and grief, being a coherent thread throughout the site. This was postulated to help participants develop a relationship with the site. Flexibility of choices was also deemed important. Grief support has some common elements and principles, but to make the site more individually tailored, participants were encouraged to dip in and out, finding their own way through the materials. This was exemplified in the intervention’s name, “My Grief My Way”, which was arrived at through PPI engagement. Rather than the material being presented in a linear fashion, like a traditional book, the metaphor of a ‘choose your own adventure’ novel highlighted that participant could pick and choose elements that they found appealing or relevant to their own situation.

The use of a neutral, natural green colour palette, and abundant nature imagery was chosen to be soothing and to enhance nature connection. Other images presented a diversity of people, with signals of different ethnicity, age, gender and sexual orientation, again designed to maximise inclusivity and belonging. All these elements were designed to contribute to a sense of psychological safety.

Finally, a number of other elements of psychological theory and bereavement support were incorporated into the website. These included educational interventions, links to practical supports (e.g., financial advice), and other psychological concepts such as self-compassion (Neff, 2023), Continuing Bonds (Klass & Steffen, 2018) and dual process theory (Stroebe & Schutt, 1999).

*Volunteer Support*

One aspect of the ‘Intervention Components’ was initially volunteer support. During the iterations of the logic model this came to be seen as increasingly its own factor, based upon the feedback from the evaluation. Given that some participants elected to use My Grief My Way without a volunteer supporter (i.e., entirely self-directed), we separated out the intervention components that were thought to underpin the online intervention and presented the intervention component associated with volunteer support separately. Volunteer support provided an actual supportive relationship with another human being, it allowed for emotional expression, active listening and understanding. In addition, the support volunteers had received training in the ACT model and were able to use that to try out ACT skills delivery and reinforcement of the clients’ efforts. They were also able to troubleshoot use of techniques, clarify elements of understanding of the concepts, as well as general help in problem solving.

*Change Mechanisms*

In earlier iterations of the logic model, Psychological Flexibility (OPEN, AWARE, ENGAGED Skills) was considered to be the primary change mechanism. This perception of the potential change mechanisms expanded via synthesis of PPI perspectives, expert perspectives, service provider and volunteer supporter perspectives and the mixed methods evaluation, to include a broader range of potential change mechanisms. Psychological flexibility was still considered a core mechanism. In addition, enhanced emotion regulation through increased distress tolerance, ability to express emotions, and to self soothe or receive comfort from others was also important.

Processes of perspective taking, being able to connect with what matters now after loss and appreciating that the pain of grief is related to our attachment with the deceased were collectively considered to be forms of meaning making / reconstruction. Processes of normalisation and optimism were also seen as potential meaning-based change mechanisms.

Finally, many participants in the evaluation spoke of the website as making them feel less alone. This led us to enhance the position of social support as an important change mechanism. This was particularly so for those who engaged in My Grief My Way with a trained support volunteer.

These change mechanisms are considered to be in a reciprocal relationship with the intervention components: the intervention components attempt to operationalise the change mechanisms. Similarly, both of these are in a reciprocal relationship with the indicators of higher grief support need, as the change mechanisms and intervention components try to target the pathways that are encapsulated in the indicators. For example, overwhelming emotion and avoidance are targeted by awareness and openness skills training, via video presentation, metaphor and worksheet. These reciprocal relations are illustrated by the arrows between boxes, conveying the tight interplay between elements in the design of My Grief My Way.

*Outcomes*

Individual short-term outcomes included greater emotion tolerance and regulation, and increase in active forms of coping (i.e., non-avoidance, non-withdrawal, healthy engaging in activity, relationships, hobbies, work etc), seeking out adaptive social support, talking to other people more openly about grief. Increased psychological flexibility and wellbeing were also key short-term outcomes.

On an individual level, long term outcomes were considered to be doing more of what matters in life, either reconnecting with relationships or establishing or deepening new relationships and investment in self-care.

As well as individual participant outcomes, it was anticipated that there would be significant outcomes for the bereavement support volunteers who had received training in ACT as part of this study. These outcomes were postulated to be enhanced skills and tools, their own increased psychological flexibility and greater confidence in providing bereavement support. A publication reporting the process of training and the outcomes for the volunteers is currently in preparation.

*Impact*

Impact refers to broader changes that are expected to result from the intervention, organised by three subtypes of impact. At an individual level, we anticipated that My Grief My Way would have a beneficial impact due to increased availability of evidence-based support, as well as greater choice of how to access bereavement support. We anticipate that the self-directed aspect of My Grief My Way will likely lead to users experiencing increased autonomy and agency, due to their being able to use it in a manner, time and frequency that suits their needs.

For organisations that offer bereavement support, it is postulated that adoption of My Grief My Way would lead to greater reach, serving more people in an effective and cost-effective manner, as well as providing an additional service offering or pathway. This would be particularly relevant for people who would prefer not to talk to a counsellor of volunteer, who do not need that level of intervention, or are waiting to receive a more intensive support intervention.

In terms of broader impact at a societal level, an intervention like My Grief My Way may help improve public grief literacy (e.g., Selman, 2024). This could help to shift misconceptions about bereavement and ensure that grief support needs are more widely recognised. The website being independent of statutory mental health services may also help to de-pathologize / de-medicalize grief. Finally, the themes of ‘no correct way to grieve / many paths through the forest’ may help to change public perception of grief as a linear and stage like process, to what is a more fluid, active, circular and interwoven experience.

**Discussion**

This paper describes the development of a logic model to guide My Grief My Way, an online support intervention for people with moderate bereavement support needs. It provides an example of the use of logic models in intervention design. The logic model transparently outlines the theoretical foundations and research evidence around risk factors and processes that contribute to higher bereavement support needs, and indicators of such needs that individual clients may present. Change mechanisms and intervention components that operationalise these were tightly organised in relation to these postulated risk factors, indicators and processes. A range of outcomes and impacts were forecast at individual, organisational and societal levels at both short and long terms.

One of the most useful aspects of this logic model, and logic models by extension, is their ability to integrate a very wide range of sources of information and multiple perspectives into a coherent synthesis. For instance – the contributions of distinct psychological theories, principles and models, behaviour change theories, theories of grief mechanisms, evidence about grief trajectories, the experiences of bereavement support providers, the expertise of grief researchers and the experiences of people with lived experience of a wide range of bereavements are all balanced within the logic model.

The process of intervention development in this study was fundamentally relational. In co-producing an intervention with these multiple stakeholders, we had to engage well with a broad range of perspectives and understandings of the phenomenon of interest. Creating a logic model not only captured these diverse perspectives and sources of knowledge, it helped the team to communicate with professional interdisciplinary and lay members and to receive feedback. The demonstration of this as an iterative process allowed these stakeholders to see how their knowledge and expertise was being used to shape the understanding of the intervention and its mechanisms, creating a high level of ownership of the logic model.

The logic model was also fundamentally pragmatic – it led to intervention components that closely matched change mechanisms and intervention targets. These targets are the indicators of higher support needs. These indicators are framed as processes (e.g., avoidance, existential concerns), which the intervention components and change mechanisms target. This leads to tight and coherent links between understanding the context of the ‘problem’ and how the intervention will alter that.

A further aspect of the iterative nature of the development of a logic model is the need for the research team to remain open and flexible as they engage with stakeholders. The logic model for My Grief My Way evolved over time. This is most evident in our understanding of change mechanisms, beginning with psychological flexibility but then developing to incorporate a broader range of mechanisms. Some of these mechanisms could still be understood within the umbrella of psychological flexibility. An example is that the mechanism of emotion regulation could also be understood as greater openness / acceptance of emotion, allowing it to be processed. Incorporating a more diverse set of understandings gives the advantage of the logic model being more appealing and intuitive to a broader population of practitioners and service providers which is a pragmatic and utilitarian outcome.

Iteration came not only through incorporating multiple stakeholders’ viewpoints, but also as we gathered data from the mixed method evaluation of the My Grief My Way intervention that flowed from the logic model. As we heard the comments and experiences of the bereaved participants and bereavement support volunteers, we had new insights and understandings of the intervention, its components and its theoretical processes. Logic models should therefore not be seen as static entities. They are fluid and responsive to feedback from the interventions that they guide.

*Implications for theory*

Given the highly integrative nature of this logic model, there are implications for theory. For example, there are four main theories that are represented in this logic model and a number of other concepts. The theories are ‘Psychological Flexibility’ (Hayes et al., 2006), Dual Process Theory (Stroebe & Schut, 1999), Meaning Making (Niemeyer et al., 2010) and Continuing Bonds (Klass & Steffen, 2018). Each of these comes from a distinct historical and interventional context and yet the logic model integrates these well. Does this result in the creation of a new theory or model of effective or ineffective grieving? Our perspective is that it does not, but that each of the grief theories give guidance about important psychological processes that are hallmarks of effective or ineffective grieving, and that the Psychological Flexibility theory gives general guidance about how human beings respond to unpleasant or unwanted thoughts, emotions, memories and sensations. The integration provided by the logic model suggests that psychological flexibility and its component skills trainings of open, aware and engaged are pragmatic tools that operationalise and support the predictions outlined by Dual Process Theory (e.g., allowing oneself to go between loss-oriented and restoration-oriented activities) with awareness, pragmatic focus on consequences and sources of influence on behaviour. Similarly, Psychological Flexibility gives concrete strategies of how to support a continuing bond with a lost person, how to conceptualise that relationship as an ongoing valued commitment and actions, as well as promoting awareness of consequences, allowing a person to make their own mind up as to whether their continuing bond is healthy and effective or dysfunctional and maladaptive.

Other concepts integrated into the logic model include normalisation, education and information. These ideas can be incorporated into another theory in My Grief My Way, that of meaning making (Niemeyer et al., 2010). Through normalisation and education, previously threatening experiences can come to be understood as part of a process. Psychological flexibility, skills of awareness and tracking can help people to directly assess their own meanings and the consequences of being guided by different meanings. Values work can help to create new meaning and new perspectives, altering these phenomena from overwhelming and threatening to understandable and contained. With such processing, emotions can be experienced and do not need to be avoided. Again, the integration of these concepts does not need to result in a new theory, but show how multiple perspectives can be synthesised, leading to a fuller and more practical understanding of grieving and how to support people to live well after bereavement.

*Implications for practice*

The logic model can be used by bereavement support organisations in assessing bereavement support needs, alongside other assessment tools and strategies. It suggests differing levels of support need and transparently outlines indicators of need. Organisations may benefit from developing alternative service offerings, varying in intensity according to need. These could be self-directed or volunteer supported, with the My Grief My Way intervention being part of that offering.

From an individual level, volunteers, therapists, counsellors and psychologists who support people after bereavement could incorporate My Grief My Way into their practice, using it to structure support, offer homework strategies and to reinforce the work happening in sessions. This could also be used to support bereavement group work.

*Strengths and limitations*

Strengths of the logic model include the interdisciplinary integration of multiple theoretical perspectives, the co-production approach, and the synthesis of multiple sources of information in a coherent and accessible way.

In terms of limitations, the logic model has arisen out of dialogue and relationship, as well as evidence and theory, and the exact evidence that underpins every aspect of it may be difficult to precisely pinpoint. A further limitation is that we began from the point of view that ACT and Psychological Flexibility would be a useful framework for this project. It is possible that had we started from a more neutral set of experiences and learning histories that we may not have oriented this work towards that theoretical domain. However, it is a framework that has proved to be both utilitarian and rich in empirical evidence across a wide range of phenomenon over the years (e.g., Gloster at al., 2020), and therefore was a natural starting point for this endeavour.

A final limitation is that the particular perspectives and histories of our partners, both on the steering group, and in the PPI group undoubtedly influenced the logic model that we produced. We made considerable efforts to engage and recruit a diversity of voices, including diversity of gender, ethnicity, sexual orientation, and multiple types of losses, and experiences of bereavement support and grieving. However, a different group of stakeholders and different perspectives may have emphasised or de-emphasised certain features of the logic model.

*Future Research*

The development stage for My Grief My Way is now complete, with the creation of the logic model and piloting to refine the intervention. Future research that tests My Grief My Way in a larger population, with a focus on intervention implementation in routine settings, and that measures efficacy relative to providing no intervention or information only is needed to fully test the role that My Grief My way could play in increasing access to evidence based bereavement support. Logic models are subject to continual evolution, and as future research evidence is gathered so this model may be refined.

The logic model also provides a useful starting point from which to develop further logic models, such as an implementation model outlining pathways to adoption of My Grief My Way in routine service settings across public and third sector organisations. In addition, the logic model can be adapted to also guide the development of studies that adapt My Grief My Way for other populations such as children and young people. Other populations that could benefit from an adapted version of My Grief My way (and an associated guiding logic model) are people with intellectual disability, or other forms of reduced cognitive ability such as in head injury or dementia.

Finally, we have been explicit that My Grief My Way and its guiding logic model is oriented towards people who have moderate grief support needs, but that it is not targeted at people that have the highest support needs, or the most complicated grief profiles. A future study could use the logic model presented here to explore adaptations for people who meet criteria for Prolonged Grief Disorder or other expressions of grieving that are more complicated.

**Conclusion**

Whilst grieving for someone who has died is a natural phenomenon that many people appear to engage in effectively with only the support of family and friends, a substantial proportion of people do need some support after bereavement. This paper outlines the co-production of a logic model that guided an intervention development study to increase access to evidence-based support in this area via the My Grief My Way online intervention. The resultant logic model was transparent, coherent, credible and integrative and specified tight coherence between features of the context, mechanisms of change, intervention components and intended outcomes. Findings support the use of logic models as pragmatic and useful in guiding the development and evaluation of complex interventions.

**References**

Albuquerque S, Pennetta G, Coelho A, et al. (2024) Navigating grief in unprecedented times: risk factors in the wake of pandemic loss and end-of-life care. *Psychology, Health & Medicine* 0(0). Taylor & Francis: 1–14.

Aoun SM, Breen LJ, Howting DA, et al. (2015) Who needs bereavement support? A population based survey of bereavement risk and support need. *PLoS ONE* 10(3): 1–14.

Breen, LJ , Mancini, VO , Lee, SA , Pappalardo, EA , and Neimeyer, RA . Risk factors for dysfunctional grief and functional impairment for all causes of death during the COVID-19 pandemic: the mediating role of meaning. Death Stud. (2022) 46:43–52. doi: 10.1080/07481187.2021.1974666

Canny A, Finucane A, Cusinato M, et al. (in prep) Something for everyone: Views and experiences of an acceptance and commitment therapy (ACT) based training for bereavement support and the subsequent delivery of ACT to bereaved individuals.

Cardiff University (n.d.) Public Involvement in Research Impact Toolkit (PIRIT). Available at: <https://www.cardiff.ac.uk/marie-curie-research-centre/patient-and-public-involvement/public-involvement-in-research-impact-toolkit-pirit> (accessed 24 December 2024).

Cholbi M (2022) *Grief A Philosophical Guide*. Princeton, New Jersey: Princeton University Press.

Crown Copyright (2024) Understanding WCAG 2.2 - Service Manual - GOV.UK. Available at: <https://www.gov.uk/service-manual/helping-people-to-use-your-service/understanding-wcag> (accessed 24 December 2024).

Eisma, MC , and Tamminga, A . COVID-19, natural, and unnatural bereavement: comprehensive comparisons of loss circumstances and grief severity. Eur J Psychotraumatol. (2022) 13:2062998. doi: 10.1080/20008198.2022.2062998

Finucane A, Canny A, Mair, APA, et al. (2024) A rapid review of the evidence for online interventions for bereavement support. *Palliative Medicine*: 02692163241285101.

Finucane A, Gillanders D, Canny A, et al. (in prep) ‘My Grief My Way’: Development of An Online Acceptance and Commitment Training Intervention to Improve Wellbeing and Coping for People Who Have Been Bereaved.

Gibson Watt T, Gillanders D, Spiller JA, et al. (2023) Acceptance and Commitment Therapy (ACT) for people with advanced progressive illness, their caregivers and staff involved in their care: A scoping review. *Palliative Medicine* 37(8): 1100–1128.

Gillanders D, Finucane A, Hulbert-Williams N, et al. (under review) Acceptance and Commitment Therapy (ACT) for supporting people after bereavement. *Mortality*.

Gloster AT, Walder N, Levin M, et al. (2020) The Empirical Status of Acceptance and Commitment Therapy: A Review of Meta-Analyses. *Journal of Contextual Behavioral Science* 18(September). Elsevier Inc.: 181–192.

Greene J (2018) *Logic Models*. In *The SAGE Encyclopaedia of Educational Research, Measurement, and Evaluation*, Bruce Frey (Ed.), Thousand Oaks, California: SAGE Publications.

Harrop, E, Goss, S, Farnell, D, Longo, M, Byrne, A, Barawi, K, Torrens-Burton, A, Nelson, A, Seddon, K, Machin, L and Sutton, E, (2021). Support needs and barriers to accessing support: Baseline results of a mixed-methods national survey of people bereaved during the COVID-19 pandemic. Palliative medicine, 35(10), pp.1985-1997.

Harrop, E, Morgan, F, Byrne, A and Nelson, A, (2016). “It still haunts me whether we did the right thing”: a qualitative analysis of free text survey data on the bereavement experiences and support needs of family caregivers. BMC palliative care, 15, pp.1-8.

Harrop, E, Morgan, F, Longo, M, Semedo, L, Fitzgibbon, J, Pickett, S, Scott, H, Seddon, K, Sivell, S, Nelson, A and Byrne, A, (2020b). The impacts and effectiveness of support for people bereaved through advanced illness: a systematic review and thematic synthesis. Palliative Medicine, 34(7), pp.871-888.

Harrop, E, Scott, H, Sivell, S, Seddon, K, Fitzgibbon, J, Morgan, F, Pickett, S, Byrne, A, Nelson, A and Longo, M, (2020a). Coping and wellbeing in bereavement: two core outcomes for evaluating bereavement support in palliative care. BMC Palliative Care, 19, pp.1-15.

Harrop E, Medeiros Mirra R, Goss S, et al. (2023) Prolonged grief during and beyond the pandemic: factors associated with levels of grief in a four time-point longitudinal survey of people bereaved in the first year of the COVID-19 pandemic. *Frontiers in Public Health* 11. Frontiers.

Hayes SC, Luoma JB, Bond FW, et al. (2006) Acceptance and Commitment Therapy: Model, processes and outcomes. *Behaviour Research and Therapy* 44(1): 1–25.

Hayes SC, Strosahl, Kirk D and Wilson KG (2012) *Acceptance and Commitment Therapy (2nd Edition): The Process and Practice of Mindful Change*. New York: Guilford Press.

Klass D and Steffen EM (2018) Continuing bonds - 20 years on. *Continuing bonds in bereavement: New directions for research and practice*: 1–14.

Lobb, EA , Kristjanson, L , Aoun, S, et al. Predictors of complicated grief: a systematic review of empirical studies. Death Stud. (2010) 34:673–98. doi: 10.1080/07481187.2010.496686

Lundorff, M, Holmgren, H, Zachariae, R, Farver-Vestergaard, I & O’Connor, M (2017) Prevalence of prolonged grief disorder in adult bereavement: A systematic review and meta-analysis. *Journal of Affective Disorders*, 212, 138-149.

Neff KD (2023) Self-Compassion: Theory, Method, Research, and Intervention. *Annual Review of Psychology* 74, 193–218.

Neimeyer RA, Burke LA, Mackay MM, et al. (2010) Grief Therapy and the Reconstruction of Meaning: From Principles to Practice. *Journal of Contemporary Psychotherapy* 40(2): 73–83.

Neimeyer, RA , and Lee, SA . Circumstances of the death and associated risk factors for severity and impairment of COVID-19 grief. Death Stud. (2022) 46:34–42. doi: 10.1080/07481187.2021.1896459

O’Cathain A, Croot L, Sworn K, et al. (2019) Taxonomy of approaches to developing interventions to improve health: a systematic methods overview. *Pilot and Feasibility Studies* 5(1): 41.

Poxon, L, (2023) ‘Doing the same puzzle over and over again’: a qualitative analysis of feeling stuck in grief. *Bereavement. Journal of Response to Grief and Death. Vol 2*

Prigerson HG, Singer J and Killikelly C (2024) Prolonged Grief Disorder: Addressing Misconceptions With Evidence. *The American Journal of Geriatric Psychiatry* 32(5). Elsevier: 527–534.

Sanders, C. M. (1980). A Comparison of Adult Bereavement in the Death of a Spouse, Child, and Parent. OMEGA - Journal of Death and Dying, 10(4), 303–322.

Selman, L (2024) Facing death differently: Revolutionising our approach to death and grief. *BMJ,* https://doi.org/10.1136/bmj.q2815

Skivington K, Matthews L, Simpson SA, et al. (2021) A new framework for developing and evaluating complex interventions: update of Medical Research Council guidance. *BMJ*: n2061.

Stroebe M and Schut H (1999) The dual process model of coping with bereavement: Rationale and description. *Death Studies* 23(3): 197–224.

The UK Commission on Bereavement (2022) Bereavement is everyone’s business. The UK Commission on Bereavement. Available at: <https://bereavementcommission.org.uk/media/jaqex1t5/bereavement-is-everyone-s-business-full-report_1.pdf>.

Torrens-Burton, A, Goss, S, Sutton, E, Barawi, K, Longo, M, Seddon, K., Carduff, E, Farnell, DJ, Nelson, A, Byrne, A and Phillips, R, (2022) ‘It was brutal. It still is’: a qualitative analysis of the challenges of bereavement during the COVID-19 pandemic reported in two national surveys. Palliative care and social practice, 16, p.26323524221092456.

Willi N, Pancoast A, Drikaki I, et al. (2024) Practitioner perspectives on the use of acceptance and commitment therapy for bereavement support: a qualitative study. *BMC Palliative Care* 23(59): 1–13.
