# Supplementary material for: A qualitative study of bereavement support volunteers’ views and experiences on an online Acceptance and commitment therapy-based (ACT) training programme
Source: PLoS One. 2025 Dec 8;20(12):e0337321. doi: 10.1371/journal.pone.0337321 (PMC12685200; doi:10.1371/journal.pone.0337321)
Supplement: Attachment — Submitted filename: PLOS One Response to Reviewers.docx [file pone.0337321.s010.docx]

**Response to PLOS ONE Reviewers**

Reference: PONE-D-25-29547

Date: 07 October 2025

Dear PLOS ONE Editor/reviewer

Thank you for the opportunity to respond to your comments from our recent submission (PONE-D-25-29547): Views and experiences of bereavement support volunteers on an online Acceptance and Commitment Therapy-based (ACT) training programme. We believe your comments have clarified and enriched our article and so please find our responses to your comments listed below in order of receipt.

| **Academic Editor** | | |
| --- | --- | --- |
| **Comment #** | **Comment** | **Response** |
| 1. | Qualitative methodology. | Thank you. To clarify the qualitative nature of our article, we have changed the title of the manuscript to read: A qualitative study of bereavement support volunteers’ views and experiences on an online Acceptance and Commitment Therapy-based (ACT) training programme.  The qualitative methodology is explained and cited further on page five of the manuscript. |
| 2. | Trustworthiness / methodological rigour. | To meet methodological rigour and trustworthiness, we used COREQ; Consolidated criteria for Reporting Qualitative research. This 32-item checklist was developed to promote comprehensive and transparent reporting of qualitative studies that use interviews and focus groups. It guided authors on how to report details about the research team, study design, data analysis, and findings to improve the rigor and credibility (cited in page five and the completed checklist is uploaded as ‘Other’ file).  Additionally, and to limit bias in our findings, three researchers independently coded two identical transcripts (one focus group and one individual interview) using a combination of inductive coding and deductive coding. Initial themes were organised into a set of categories to form a series of refined category matrices. Through in-depth team discussions, consensus was reached on final themes, to which the remaining study data were coded, resulting in systematically structured outputs of summarised data. These statements are detailed on page nine of the manuscript. |

| **Journal Requirements** | | |
| --- | --- | --- |
| **Comment #** | **Comment** | **Response** |
| 1. | PLOS ONE style requirements. | Thank you. These have been checked and are in order. |
| 2. | Title page within main document. Authors and affiliations included in the manuscript file itself. | The new title is included in main document as are all authors and affiliations. |
| 3. | Funding. | The funders had no role in study design, data collection and analysis, decision to publish, or preparation of the manuscript. |
| 4. | Restrictions to data sharing.  a. Explain why ethical or legal restrictions on sharing a de-identified data set.  b. If no restrictions, upload minimal anonymised data set necessary to replicate study findings to a stable public repository with URLs or accession numbers. | Anonymised qualitative data not published within this article may be made available by reasonable request from qualified investigators. Please direct requests to the Health in Social Science Research Ethics committee at the University of Edinburgh: ethics.hiss@ed.ac.uk.  a. Data from this small sample of participants were collected from two organisations. Within these organisations, participants were often colleagues and as such, published de-identified data could be at risk of identification.  b. Although we have not provided a full data set, we believe we have offered sufficient data in terms of baseline characteristics and study themes (tables 2 and 3) alongside an extensive range of qualitative quotations to help replicate study findings (pages 12-24). |
| 5. | Contact for access to anonymised data. | Please direct requests for access to anonymised data to the School of Health in Social Science, Research Ethics committee at the University of Edinburgh: ethics.hiss@ed.ac.uk. |
| 6. | Full ethics statement to be included in Methods section. | This statement has been added to the Methods section (page nine). |
| 7. | Add abstract to manuscript. | Abstract has been added to manuscript after the title page (page 1-2). |
| 8. | Supporting Information S1-S4, copyrighted images. | We believe the images used in S1-S4 are of a public nature, but to err on the side of caution, we have removed all images where we could not obtain copyright.  The training slides were created and are owned by Dr David Gillanders. He has completed the ‘Content Permission Form’ and this is uploaded as ‘other file’.  I’m afraid we do not have a link to the files (as per the Content Permission Form instructions) but trust the uploaded PDFs as ‘other file’ satisfies this request.  For the images that remain, the additional requirement to include:  “Reprinted from [ref] under a CC BY license, with permission from [name of publisher], original copyright [original copyright year]” has been completed. |
| 9. | Supporting Information S2 includes an image of a patient/participant. | The image has been removed. |
| 10. | Reviewer may request citation of previously published work. | No action required at this time. |
| 11. | Review references to ensure complete. | References checked and are complete and correct. |

| **Reviewers # 1 & 2 General Comments** | | |
| --- | --- | --- |
| Comment # | Comment | Response |
| 1. | Technically sound piece of scientific research. | No action required |
| 2. | Has statistical analysis been performed appropriately and rigorously? I don’t know. | This is a qualitative study therefore no statistical analyses were conducted. We have amended the title of our article to make this clearer to the reader. |
| 3. | Data available? Yes. | No action required. |
| 4. | Manuscript presented in an intelligible fashion and in English? Yes. | No action required. |
| **5a. Specific Comments by Reviewer #1** | | |
|  | Comments by Reviewer no1:  Acceptance and Commitment Therapy-based Training and Acceptance and Commitment Training are used interchangeably.  Are findings statistically significant?  Number is low and mostly female.  Does support received over the telephone, online or in person affect the outcome?  Did holidays have an impact on withdrawals? | Thank you for pointing this out. We have amended the abstract to meet consistency.  This is a qualitative study therefore no statistical analyses were conducted. We have amended the title of our article to make this clearer to the reader.  One of the key factors in qualitative research relates to what is termed ‘data-rich’ cases providing an in-depth understanding of a particular phenomenon. This means that the number of participants is not as important as you would find when adhering to a specific number of participants in quantitative research and statistical generalisability.  The study involved volunteers operating in bereavement services across the UK which are predominately female. We feel our sample is representative of this typical employment sector demographic.    This question was not examined as the qualitive research was focused on development and implementation rather than a study design that would investigate such variables (i.e., RCT).  Again, this question was not examined as above. |
| **5b. Specific Comments by Reviewer #2** | | |
| 1.  2.  3.  4. | Requested additional limitations?  Process of how clients were matched to volunteers not rigorously detailed.  Volunteer perceptions only. No client-reported outcomes or experiences.  No systematic evaluation of volunteer understanding of ACT before and after training. Lacking objective skill assessments (via role-play, observed sessions) weakens conclusion on training effectiveness.  Potential Hawthorn effect? | Thank you. These processes were based on local organisational procedures therefore, we can agree. We have added a statement to reflect this limitation (page 27).  It was not the aim of this article to explore client-reported outcomes or experiences.  It was not the aim of this research to demonstrate training effectiveness. We were able to document training experience, acceptability and barriers to inform future implementation trials.  The stage of this study in the research development and implementation process is not one in which significant control is required. Therefore, yes, it could be that any involvement in research could have been regarded positively. The stage of development of a qualitative intervention development study does not seek rigorous control, but instead engagement and co-production. |
